# Single-cell and spatial transcriptomics reveals an anti-tumor neutrophil subgroup in microwave thermochemotherapy-treated lip cancer

**DOI:** 10.1038/s41368-025-00366-8

**Published:** 2025-05-13

**Authors:** Bingjun Chen, Huayang Fan, Xin Pang, Zeliang Shen, Rui Gao, Haofan Wang, Zhenwei Yu, Tianjiao Li, Mao Li, Yaling Tang, Xinhua Liang

**Affiliations:** 1 State Key Laboratory of Oral Diseases & National Center for Stomatology & National Clinical Research Center for Oral Diseases & Department of Oral and Maxillofacial Surgery, West China Hospital of Stomatology, Sichuan University, Chengdu, China; 2 State Key Laboratory of Oral Diseases & National Center for Stomatology & National Clinical Research Center for Oral Diseases & Department of Oral Pathology, West China Hospital of Stomatology, Sichuan University, Chengdu, China; 3 University of Electronic Science and Technology of China, Chengdu, China

**Keywords:** Oral cancer, Cancer genomics, Cancer microenvironment, Cancer therapy

## Abstract

Microwave thermochemotherapy (MTC) has been applied to treat lip squamous cell carcinoma (LSCC), but a deeper understanding of its therapeutic mechanisms and molecular biology is needed. To address this, we used single-cell transcriptomics (scRNA-seq) and spatial transcriptomics (ST) to highlight the pivotal role of tumor-associated neutrophils (TANs) among tumor-infiltrating immune cells and their therapeutic response to MTC. *MNDA*^+^ TANs with anti-tumor activity (N1-phenotype) are found to be abundantly infiltrated by MTC with benefit of increased blood perfusion, and these TANs are characterized by enhanced cytotoxicity, ameliorated hypoxia, and upregulated *IL1B*, activating T&NK cells and fibroblasts via *IL1B*-*IL1R*. In this highly anti-tumor immunogenic and hypoxia-reversed microenvironment under MTC, fibroblasts accumulated in the tumor front (TF) can recruit N1-TANs via *CXCL2*-*CXCR2* and clear N2-TANs (pro-tumor phenotype) via *CXCL12*-*CXCR4*, which results in the aggregation of N1-TANs and extracellular matrix (ECM) deposition. In addition, we construct an N1-TANs marker, *MX2*, which positively correlates with better prognosis in LSCC patients, and employ deep learning techniques to predict expression of MX2 from hematoxylin-eosin (H&E)-stained images so as to conveniently guide decision making in clinical practice. Collectively, our findings demonstrate that the N1-TANs/fibroblasts defense wall formed in response to MTC effectively combat LSCC.

## Introduction

Lip squamous cell carcinoma (LSCC) is a type of head and neck squamous cell carcinoma (HNSC), that primarily affects the lower lip, with rare metastases and a favorable prognosis but severe lip aesthetic damage.^1,2^ Surgery is still used to treat LSCC; however, aesthetic demands are often challenging. Professor Mao Zu-yi from our team initiated clinical research on microwave thermochemotherapy (MTC) for the treatment of LSCC in the late 20th century, discovering that microwaves can heat local tumor tissues to 42-45°C, selectively killing cancer cells while causing no harm to normal tissues.^3^ Moreover, in a randomized controlled trial of 329 patients with soft tissue sarcoma, the combination of neoadjuvant chemotherapy and local thermotherapy was found to effectively treat tumors and increase 5-year survival rates by 11.4%.^4^ Microwave thermotherapy is also being explored as an adjuvant treatment for refractory or recurrent malignant germ cell tumors (adjuvant to surgery) as well as for recurrent breast cancers (adjuvant to radiotherapy), with favorable outcomes.^5,6^ These findings imply that MTC may soon become part of the standard of care for LSCC.

However, because MTC does not benefit all LSCC patients, the outstanding question is determining the underlying mechanisms and therapeutic response of LSCC to MTC. To date, thermotherapy has been shown to cause immunogenic cell death (ICD), leading to the release of damage-associated molecular patterns (DAMP) to recruit immune cells to infiltrate tumors and mobilize antigen-presenting cells, with the help of increased vascular permeability and chemokines, resulting in the reversal of the immunosuppressive tumor microenvironment (TME).^7^ The secretion of thermal-mediated cytokines (such as IL-6 and IFNγ) in the TME consequently boosts the cytotoxicity of T cells against cancer cells.^8^ However, elucidating the molecular mechanisms and potential markers for predicting MTC treatment response is not yet simple.

Single-cell analysis has provided critical insights into the various tumor immune microenvironment (TIME) patterns and underlying mechanisms.^9^ However, only a few studies have investigated pre- and on-treatment samples of the same patient, owing to the difficulty in obtaining biopsy specimens from patients undergoing treatment. Therefore, to explore the potential mechanisms of therapeutic response to MTC, we performed single-cell transcriptome (scRNA-seq) and spatial transcriptome (ST) on pre- and on-treatment biopsies of two patients with LSCC who were receiving MTC treatment (MT, pingyangmycin, and methotrexate).

Here, we identified remarkable immune cell infiltration during MTC at the tumor front (TF). Analysis of the functions of T and NK cells revealed that immune exhaustion was improved. However, effector/cytotoxicity-associated genes were not markedly expressed, suggesting that T cell-mediated anti-tumor effects may play a partial role in treatment. This may also explain why the overall HNSC response rate to immune checkpoint blockade (ICB) is still low.^10^ Our attention was focused on myeloid cells, and we found that the proportion of neutrophils was abundant and increased significantly under MTC. We then focused on TF, distinguished by significant enrichment of neutrophils and extracellular matrix (ECM), and conclusively established neutrophils as crucial contributors to the anti-tumor mechanisms of MTC.

## Results

### Single-cell and spatial transcriptomics profiling of LSCC under MTC

**Fig. 1:**
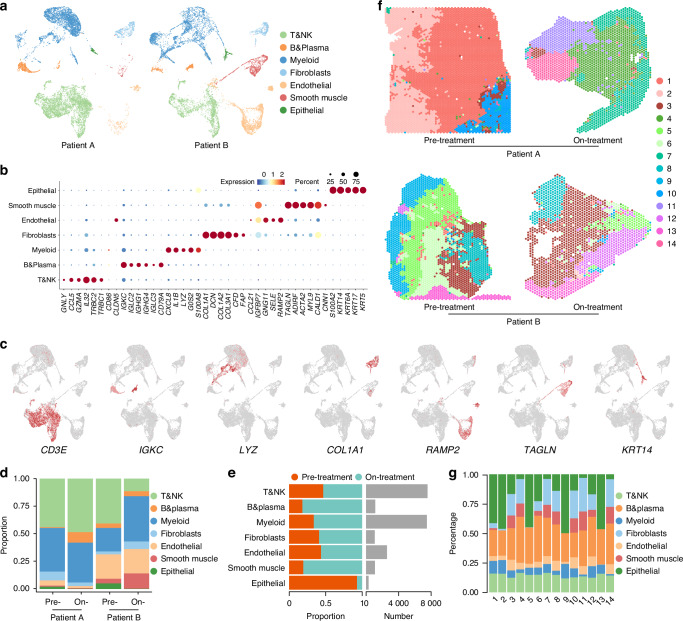
Single-cell and spatial transcriptomics atlas of LSCC. **a** UMAPs of 21 108 cells from patients A and B, including pre- and on-treatment. **b** Dot plot showing average expression of known markers in indicated cell clusters. **c** UMAPs showing expression levels of selected marker genes in each cell type. **d** Bar plots showing proportions of 7 major cell types in each samples. **e** Bar plots showing the distribution of 7 major cell types before and during treatment (left panel) and the number of cells of each cell type (right panel). **f** Unbiased clustering of ST spots of four samples. **g** Bar plots showing tissue distribution of 7 major cell types

Over the last 5 years, our group has treated 37 LSCC patients with MTC. After the entire treatment, 25 patients had satisfactory therapeutic effects, and 18 had no recurrence after follow-up (Fig. S1). To monitor intratumor changes in LSCC patients under MTC, we processed four fresh samples from two patients before and during treatment, with their informed consent. First, we performed scRNA-seq and ST analyses using the 10X genomics platform (Fig. S2a). In the scRNA-seq analysis, we filtered and preprocessed 23 366 cells (Table S1), retaining 21 108 cells with a mean depth of 56 643 reads per cell. The data were then visualized by unified modal approximation and projection (UMAP), and seven major cell types were identified in two patients (Figs. 1a, S2b). T&NK cells (*n* = 7 516) were labeled by *CD3E* and *TRBC1*, myeloid cells (*n* = 7 422) mainly expressed *LYZ* and *CD86*, and *IGKC* and *CD79A* marked B&plasma cells (*n* = 1 122). In addition, fibroblasts (*n* = 1 068) were identified by *COL1A1* and *FAP*, endothelial cells (*n* = 2 561) were positive for *RAMP2* and *CLDN5* expression, smooth muscle cells (*n* = 1 100) were defined by *TAGLN* and *CNN1*, and epithelial/cancer cells (*n* = 319) were labeled by *KRT14* and *KRT17* (Fig. 1b, c). Both pre- and on-treatment samples from patients A and B showed all seven major cell types with no cell cluster bias. After measuring the proportion, distribution, and number of the seven cell types in the four samples, we were concerned about a significant decrease in the proportion of epithelial/cancer cells as well as the highest number of T&NK cells and myeloid cells during the treatment, implying that MTC may lead to ablation of LSCC cells through an anti-tumor immune response (Fig. 1d, e).

The scRNA-seq analysis focused on the heterogeneity of all cells within the tumor tissue, but lacked information about their spatial location; therefore, we also performed spatial transcriptomics. In the ST analysis, we obtained 4 590, 1 937, 2 600, and 1 640 spots with median depths of 3 518, 10 655, 5 417, and 6 767 UMIs/spot and 1 730, 2 644, 1 814, and 2 154 genes/spot from the four samples, respectively (Table S2). First, all the spots were classified into 14 clusters after unbiased clustering (Figs. 1f, S2c, d). Then, the enrichment score and proportion of all major cell types were calculated for each spot cluster using the cell type markers derived from scRNA-seq data (Figs. 1g, S2e). Our results showed that the clusters were consistent with the histological annotations (Figs. 1f, g, S3), validating the clustering across the four samples and confirming the ability to distinguish different spatial regions within the sections based on ST gene expression.

### Characterization of the TME and cell type mapping in LSCC under MTC

**Fig. 2:**
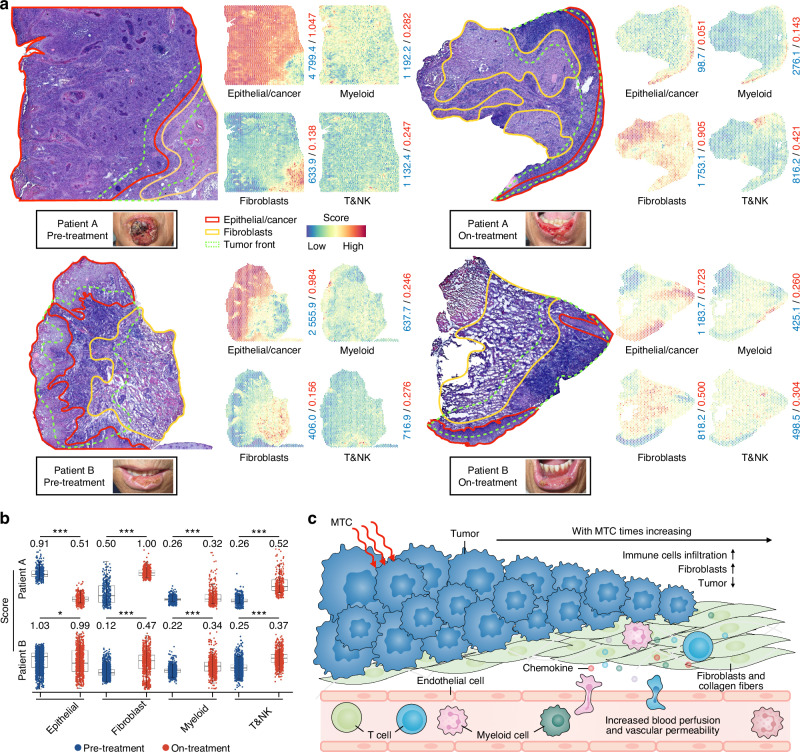
TME atlas of LSCC. **a** H&E maps of four samples with delineated areas of tumor cells, fibroblasts, and tumor front. Spatial distribution of epithelial/cancer cells, fibroblasts, T&NK cells, and myeloid cells (the right side of the H&E diagram). The blue numbers on the right side of the ST plot indicate the total infiltration score for each cell type throughout the image, and the red numbers indicate the mean score of every spot. Pre- and on-treatment labial photographs of patients A and B were shown. **b** Box plots showing the infiltration scores of different cell types in TF of pre- and on-treatment, with specific scores labeled. *P < 0.05, ***P < 0.001. **c** Schematic diagram of TME in LSCC under MTC. The mean values across groups were compared using a 2-tailed Student’s *t*-test

During the medical procedure, the LSCC lesions of patients A and B remarkably diminished with apparent elasticity on palpation after undergoing five times (half a course) of MTC (Fig. 2a). Biopsies and hematoxylin-eosin (H&E) staining were performed on four samples, including pre- and on-treatment, and the distinct histological features on the slides were annotated. Three central regions were defined in both pre- and on-treatment sections: cancer, fibroblasts, and TF areas, consisting primarily of immune cells and cancer cells, with fewer cancer cells and more normal epithelial cells in the on-treatment sections (Figs. 2a, S4a). Using ST, all cell types were mapped, revealing that their spatial distribution was similar to that of the outlined H&E map (Figs. 2a, S5a). Analysis of the scRNA-seq data showed reduced G2/M phase scores in epithelial/cancer cells from the on-treatment samples (Fig. S5b). Moreover, gene function analysis in epithelial/cancer cells revealed that upregulated genes were associated with promoting immune cell activation and cytokine release, whereas downregulated genes were involved in cell migration (Fig. S5c). These results suggested that MTC reduced the proliferation and migration of epithelial/cancer cells. Fibroblasts are interstitial cells implicated in numerous studies as a critical cell type for tumor progression.^11^ scRNA-seq analysis identified 12 interstitial cell subtypes, including fibroblasts, endothelial cells, and smooth muscle cells (Fig. S5d, e). Gene ontology (GO) enrichment analysis of these interstitial cells revealed that fibroblasts are essential for facilitating immune cell chemotaxis, regulating myeloid differentiation, promoting cytokine release, and regulating T-cell activation (Fig. S5f).

Next, we focused on the cellular characteristics of the TF. ST was used to examine its cellular components (681, 612, 1 329, and 698 spots were detected from the four samples, respectively), which included epithelial/cancer cells, fibroblasts, T&NK cells, myeloid cells, B&Plasma cells, endothelial, and smooth muscle cells (Figs. S6, S7a). Notably, the spatial infiltration of fibroblasts, T&NK cells, myeloid cells, B&Plasma cells, endothelial, and smooth muscle cells in the TF areas was obviously increased in the on-treatment sections compared to pre-treatment (Figs. 2b, S7b). Although B&Plasma cells exhibited increased spatial infiltration in TF, they were significantly enriched in both tumor and non-tumor spaces and did not demonstrate tumor-related specificity. Therefore, B cells were not a focus of this study. Moreover, we analyzed differentially expressed genes (DEGs) within the TF areas (on-treatment vs. pre-treatment) (Fig. S8a–c). GO enrichment analysis of DEGs overexpressed in the TF regions of both patients showed that they were mainly enriched in promoting extracellular matrix organization, leukocyte migration, leukocyte-mediated immunity, and cell chemotaxis, which was consistent with our observation of increased infiltration of fibroblasts and immune cells (Fig. S8d). Multimodal intersection analysis^12^ (MIA) further confirmed the three outlined parts in the H&E map (Fig. S8e), which also supported the ability of ST to identify spatial regions of different cell subtypes. In addition, we noted increased blood circulation and vascular permeability in endothelial cells and smooth muscle cells (Fig. S8f, g, Table S3). This was based on the hypothesis that MT can improve vascular permeability and chemokines to shape the TME.^13^ The combination of the patient’s clinical manifestations and these findings suggested that MTC can effectively destroy LSCC cells and increase blood perfusion, vascular permeability, fibrosis, and cellular chemotaxis in the TF, ultimately leading to increased immune cell in filtration (Fig. 2c).

### T&NK cells in the LSCC microenvironment under MTC

**Fig. 3:**
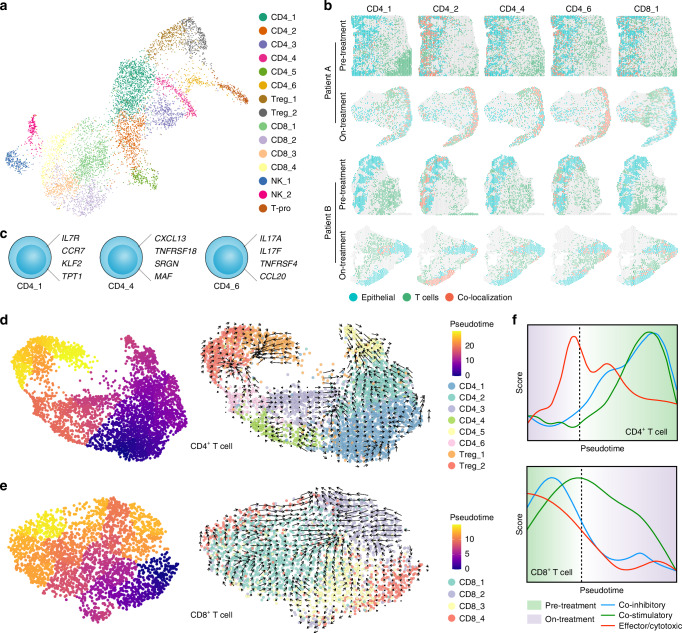
T&NK cell status in LSCC. **a** UMAP of T&NK cells from patients A and B. **b** Spatial distribution of T cells and epithelial/cancer cells. Blue dots represent epithelial/cancer cells, green dots represent different subtypes of T cells, and red dots represent the co-localization of epithelial/cancer cells and T cells. **c** Diagram of CD4^+^ T cell subpopulations. **d** UMAP showing pseudotime inference and RNA velocity analysis of CD4^+^ cells. **e** UMAP showing pseudotime inference and RNA velocity analysis of CD8^+^ cells. **f** Plots illustrating the relationship between pseudotime and treatment phase, and smooth curves showing the score of co-inhibitory, co-stimulatory, and effector/cytotoxic of CD4^+^ and CD8^+^ cells

T&NK cells were significant in the LSCC infiltration microenvironment following MTC. We identified 15 subpopulations of T&NK cells to investigate their immune activity and discovered the proportions of CD4_1, CD4_2, CD4_4, CD4_6, and CD8_1 T cells increased during treatment (Figs. 3a, S9a, b). Moreover, we investigated the spatial co-localization of epithelial/cancer cells and all subtypes of T&NK cells using ST analysis (Figs. 3b, S10a). During the treatment, there was an increase in the infiltration of 10/15 and 11/15 T&NK subpopulations in the epithelial/cancer areas of patients A and B, respectively (Fig. S10b). Furthermore, CD4_1, CD4_4, and CD4_6 T cells were significantly increased in both epithelial/cancer areas of patients, implying that MTC may facilitate the accumulation of CD4^+^ T cells in the TF areas. In addition, the gene signatures for these CD4^+^ T cell subsets were characterized: CD4_1 was abundant in effector memory genes (*IL7R*, *CCR7*, and *RPS8*), CD4_4 was abundantly expressed in follicular helper T effector genes (*CXCL13* and *TNFRSF18*), and CD4_6 was characterized by T helper 17 (Th17) cells (*IL17A* and *IL17F*) (Figs. 3c, S9a).

To investigate the evolutionary dynamics of T cells, we performed a pseudotime trajectory analysis, which revealed that CD4^+^ T cells from the on-treatment samples were located in the anterior segment of the trajectory and terminated upon pre-treatment. In contrast, CD8^+^ T cells of the pre-treatment group were located in the anterior segment of the trajectory and ended with the on-treatment, which was also confirmed by RNA velocity results (Figs. 3d, e, S11a, b). sAlong the pseudotime, we found a significant decrease in the expression of co-inhibitory markers (such as *CTLA4* and *TIGIT*, Table S4) in both CD4^+^ and CD8^+^ T cells during the on-treatment phase, which was further confirmed by their terminal exhaustion scores (Figs. 3f, g, S11c, d). The combined results of various analyses indicated that there were no significant changes in the levels of co-stimulatory receptors for CD4^+^ and CD8^+^ T cells, and that their effector/cytotoxic levels were attenuated during the on-treatment period (Figs. 3f, S11e, f and Table S4). The pseudotime trajectory results demonstrated that MTC alleviated the immune exhaustion of CD4^+^ and CD8^+^ T cells. However, neither exhibited a sufficiently high level of cytotoxicity to kill tumor cells. Also, the heatmap of the co-inhibitory, co-stimulatory, and effector/cytotoxic gene expression patterns in pre- and on-treatment samples yielded comparable results (Fig. S12a). However, higher infiltration of CD4_1, CD4_4, CD4_6, and CD8_1 cells was closely associated with better prognosis in the HNSC cohort of The Cancer Genome Atlas (TCGA), indicating that CD4^+^ and CD8^+^ T cells possessed anti-tumor properties (Fig. S12b). Further examination of T&NK functions showed that CD4^+^, CD8^+^, proliferating Τ cells, Tregs, and NK cells might be involved in the activation of myeloid adhesion, differentiation, immunity, migration, and proliferation; thus we investigated whether myeloid cells play a more crucial anti-tumor role in MTC (Fig. 12c).

### Classification of tumor-associated neutrophils in LSCC under MTC

**Fig. 4:**
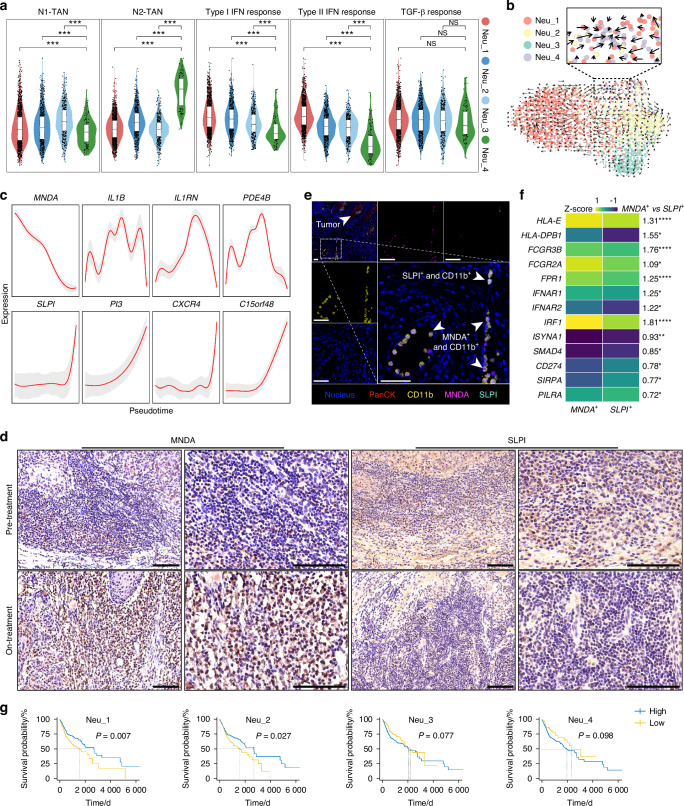
Myeloid cell characterization and N1/N2 classification of neutrophil subpopulations. **a** Violin plots of N1-TAN, N2-TAN, Type-I IFN response, Type-II IFN response, and TGFβ response scores of Neu subsets. **b** UMAP showing RNA velocity analysis of Neu subsets, with zoomed-in windows highlighting possible directional flows from Neu_2, Neu_3, and Neu_1 to Neu_4. **c** Smooth curves showing the expression of different genes along pseudotime. **d**, **e** IHC and IF fluorescence staining of MNDA and SLPI in LSCC tissues, scale bar = 100 μm. **f** Heatmap showing expression of N1- and N2-TANs related genes in *MNDA*+ and *SLPI* + TAN, and the fold difference in gene expression was annotated next to the heatmaps, *P < 0.05, **P < 0.01, ****P < 0.000 1. **g** Overall survival of Neu subpopulations infiltration in TCGA HNSC cohort. A Kaplan-Meier curve was constructed to illustrate overall survival (OS). The mean values across groups were compared using a 2-tailed Student’s *t*-test

Myeloid cell heterogeneity and roles in the treatment process were investigated, with 20 myeloid cell subsets, including dendritic cells (DC), macrophages (Mac), neutrophils (Neu), monocytes (Mon), mast cells (Mast), and myeloid-derived suppressor cells (MDSC) (Fig. 13a, b). Among all myeloid cells, DC cells increased by 0.94%, macrophages decreased by 4.11%, neutrophils increased by 4.42%, monocytes decreased by 1.75%, mast cells decreased by 0.44%, and MDSC cells decreased by 2.57% (Fig. 4b). Neutrophils were abundant in both pre- and on-treatment samples, and their components, which included four unsupervised subpopulations, changed dramatically during MTC (Figs. S13a, c, S14a). The proportion of neutrophils among myeloid cells increased from 30.6% to 34.9% during treatment, the most significant increase among all myeloid cells. In addition, Neu_1, Neu_2, and Neu_3 increased during MTC (Neu_1 increased by 1.83 fold, Neu_2 increased by 1.96 fold, and Neu_3 increased by 0.72 fold), whereas Neu_4 accounted for only 1.3% and decreased by 75% compared to pre-treatment (Fig. S13a).

Tumor-associated neutrophils (TANs) have recently received much attention and are classified as anti-tumor (N1) and pro-tumor (N2) phenotypes.^14^ In this regard, we conducted a pathway analysis. The responses of Neu_1, Neu_2, and Neu_3 to IFN were comparable, which promotes the differentiation of polymorphonuclear neutrophils (PMN) to N1, and were obviously higher than that of Neu_4. At the same time, there was no discernible difference in their responses to the TGF-β pathway, which facilitates N1 to N2 polarization (Fig. 4a). We then examined the GSE101584 from Gene Expression Omnibus (GEO) dataset and created a TAN N1/N2 phenotype gene set to score the four Neu subpopulations, revealing that Neu_1, Neu_2 and Neu_3 had similar and higher N1 phenotype scores than Neu_4, while Neu_4 had the highest N2 phenotype score (Figs. 4a, S14b). Together, it suggested that Neu_1, Neu_2, and Neu_3 may belong to N1, and Neu_4 belongs to N2 phenotype. Moreover, the pseudotime trajectory of neutrophils was computed, showing that most of Neu_1, Neu_2, and Neu_3 were located at the beginning of the trajectory (corresponding to on-treatment). Simultaneously, Neu_4 was only distributed at the end of the trajectory (corresponding to pre-treatment) (Fig. S15a–c). Similarly, RNA velocity analysis revealed significant directional flow from Neu_2, Neu_3, and Neu_1 to Neu_4 (Fig. 4b). In addition, a correlation analysis between PMN gene expression^15^ and Neu subpopulation gene expression revealed stronger correlations between PMN and Neu_1, Neu_2 as well as Neu_3 (*r* = 0.41, 0.44 and 0.43, respectively) than between PMN and Neu_4 (*r* = 0.34), supporting the pseudotime trajectory results (Fig. S15d). We observed that *MNDA* (expressed in Neu_1, Neu_2, and Neu_3) gradually decreased, whereas *SLPI* (expressed only in Neu_4) increased rapidly with pseudotime (Figs. 4c, S13c). These results were consistent with the differentiation trajectory of all four neutrophil subsets, indicating that *MNDA* and *SLPI* may be markers of N1- and N2-TANs, respectively. Immunohistochemistry (IHC) staining was performed and revealed that MNDA expression increased and SLPI expression decreased in on-treatment specimens compared to pre-treatment (Figs. 4d, S16a). Double IHC staining results showed that MNDA and SLPI were expressed in different cells (Fig. S16b). Immunofluorescence (IF) staining further confirmed the colocalization of MNDA and SLPI with CD11b (a marker of neutrophils) in the TF region (Fig. 4e).

Analysis of gene expression with N1- and N2-TANs-related markers reported in published literature showed that *MNDA*^+^ TANs tend to express antigen molecules (*HLA-E* and *HLA-DPB1*), genes related to FCγR-mediated cytotoxicity (*FCGR3B*, *FCGR2A*, and *FPR1*), and IFN receptors (*IFNAR1*, *IFNAR2*, and *IRF1;*^16^) *SLPI*^+^ TANs preferred to express iNOS-related genes (*ISYNA1*), TGFβ receptors (*SMAD4*), immune checkpoints (*CD274*), and myeloid checkpoints (*SIRPA* and *PILRA*), indicating that *MNDA* and *SLPI* have theoretical support as markers of N1- and N2-TANs, respectively (Figs. 4f, S16c, and Table S4). We also discovered a novel gene, *C15orf48*, which was not previously identified in neutrophils but was strongly correlated with the N2-phenotype (Fig. 4c). Notably, higher Neu_1 and Neu_2 infiltration was significantly associated with better survival in the HNSC cohort of TCGA, whereas higher infiltration of Neu_4 may be associated with worse survival, which supports our findings that MTC increased anti-tumor N1-TANs and decreased pro-tumor N2-TANs (Figs. 4g, S16d).

### Function of N1- and N2-TANs under MTC

**Fig. 5:**
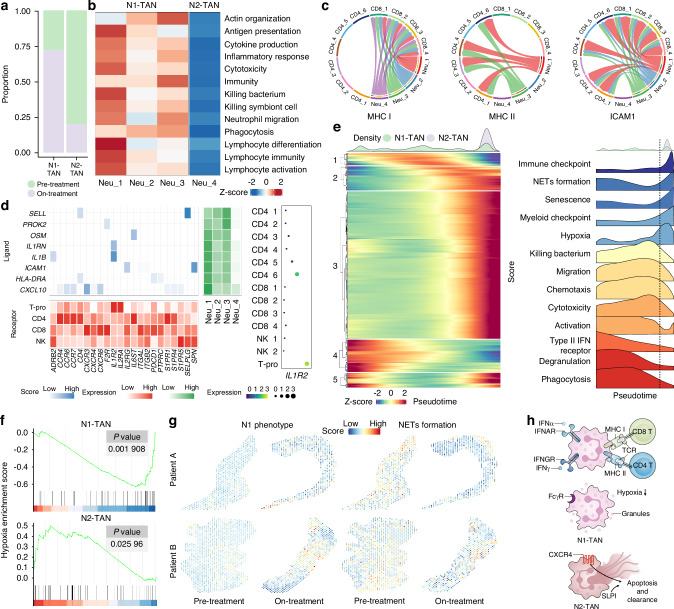
Function of N1- and N2-TANs in LSCC under MTC. **a** Bar plots showing the distribution of N1- and N2-TANs in pre- and on-treatment. **b** Heatmap showing the activation score of different pathways in N1- and N2-TANs. **c** Chord diagram showing the interactions of Neu subpopulations with T&NK cells. **d** Heatmap illustrating the ligand-receptor interaction between TANs and T&NK cells. The red heatmap below displays the expression of each receptor in receptor cells, the green heatmap on the right shows the expression of each ligand in ligand cells, and the blue heatmap in the middle illustrates the interaction strength between each receptor-ligand pair. **e** Gene expression dynamics along the TANs trajectory and genes were classified into five clusters (left panel), with a selection of function characteristics (right panel). **f** GSEA analysis showing changes in the expression of hypoxia-related genes in N1- and N2-TANs during treatment compared to pre-treatment. **g** Scores for spatial expression of N1-TAN (*TNFSF4*, *CD86*, *CD80*) and NETs-related genes (*MPO*, *PADI4*, *ELANE*) in tumor front. **h** Schematic diagram of N1- and N2-TAN functions

Neutrophils have been shown to play critical roles in tumors (both pro- and anti-tumor) through direct interactions with tumor cells or T cell-dependent pathways.^17^ Therefore, we investigated the functions of N1- and N2-TANs under MTC conditions. First, in the scRNA-seq analysis, the results showed that N1-TANs were more distributed in the on-treatment and were more competent than N2-TANs in antigen processing and presentation, migration, cytokine production, and phagocytosis, suggesting that N1-TANs can efficiently respond to external changes with migration to tumor areas and are associated with the activation of immunity (Fig. 5a, b). We then analyzed the interaction of TANs with T cells, and found that N1-TANs played an active role in MHC-I, MHC-II signaling, and *ICAM1* (Fig. 5c). Besides, ligand-receptor analysis showed that *IL1B* expression was significantly higher in N1- than in N2-TANs and was able to act on CD4_6 (defined as Th17 cells in result 2), CD8^+^ T cells, proliferating Τ cells and NK cells via *IL1B*-*IL1R2* (Figs. 5d, S17a, b), and facilitate the expression of *TNF*, *IFNG* and *IL6*, suggesting that N1-TANs could activate T&NK cells in the cytokine signaling pathway.

Further analysis of the pseudotime trajectory results revealed that the expression of immune checkpoints, NETs formation, senescence, myeloid checkpoints, and hypoxia was enriched in N2-TANs, which was consistent with N2-TANs having the reduced proportion in the on-treatment. In contrast, migration, chemotaxis, activation, cytotoxicity, IFNγR, degranulation, and phagocytosis were mainly abundant in N1-TANs, indicating that N1-TANs exhibited outstanding anti-tumor properties under MTC (Fig. 5e). Additionally, GSEA revealed that the hypoxic gene sets expression of N1- and N2-TANs was decreased and increased by MTC, respectively (Fig. 5f). These results implied that hypoxic N2-TANs became senescent during MTC, while proportionally enriched N1-TANs underwent hypoxia-reversal and activation, alleviating the hypoxic TME.

In the ST analysis, N1-TANs were significantly enriched in the TF regions of on-treatment sections compared with pre-treatment, while N2-TANs showed remarkably less infiltration (Fig. S18a, b, Table S4). Function analysis of TANs in TF showed that their chemotaxis, cytotoxicity, and degranulation were enhanced, consistent with the results of pseudotime trajectory analysis (Fig. S16c). Furthermore, the neutrophil-related functions in TF regions were analyzed, and the expression of marker genes associated with the N1-phenotype (anti-tumor effect) was significantly higher in the on-treatment group than in the pre-treatment group (Figs. 5g, S19a, b and Table S4). Besides, we specifically focused on the expression of neutrophil extracellular traps (NET)-related genes (including *MMP9* and *HMGB1*), which have been proven to promote tumor invasion by acting on extracellular matrix (ECM),^18,19^ were significantly reduced in on-treatment (Figs. 5g, S19c and Table S4). Overall, MTC enhanced the chemotaxis and ameliorated the hypoxia of N1-TANs, leading to the aggregation and enhanced cytotoxicity of N1-TANs, and promoting N2-TANs to senesce, ultimately significantly improving the anti-tumor function of TANs in LSCC (Fig. 5h).

### Intimate crosstalk between N1-TANs and fibroblasts in TF under MTC

**Fig. 6:**
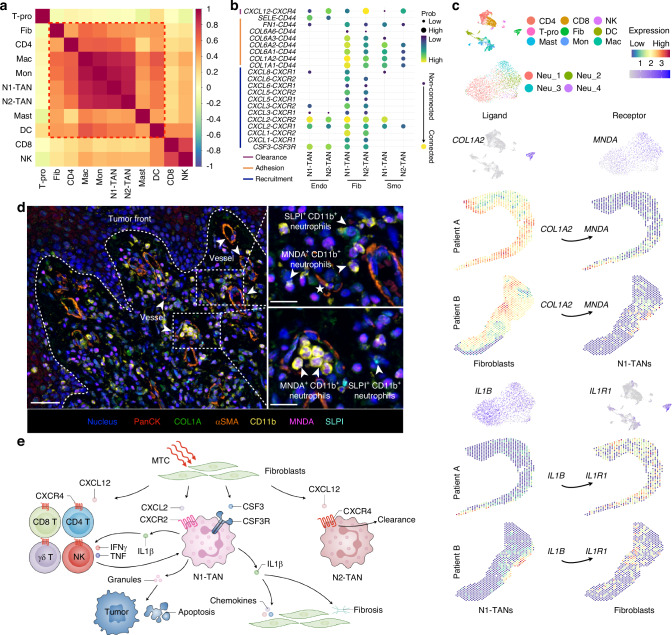
Interaction between N1-TANs and other cells in TF under MTC. **a** Correlation heatmap of fibroblasts, T&NK, and myeloid cell types in TF. **b** Dot plot of the interaction of interstitial cells (fibroblasts, endothelial cells, and smooth muscle cells) and neutrophil subclusters. **c** Single-cell and spatial expression in tumor front of selected ligands and receptors expressed by fibroblasts and N1-TANs. **d** Multiplex IHC shows the cross-talk between *MNDA*^+^ neutrophils and tumor cells. **e** Schematic diagram of crosstalk of fibroblasts and immune cells

We further explored the crosstalk between TANs and other cell types in the LSCC TME under MTC and uncovered a fibroblast-neutrophil-dominated regulatory network. Interstitial cells, primarily fibroblasts, have been described to promote cell chemotaxis and leukocyte migration and regulate myeloid differentiation. This is in line with the spatial co-localization analysis, which showed that fibroblasts co-infiltrated with neutrophils and other immune cells, consistent with the H&E map (Fig. 6a). Ligand-receptor analysis revealed that fibroblasts recruit T&NK cells, macrophages, DC cells, and monocytes via CXCL signaling and promote their adhesion through collagen signaling (Fig. S20a). Moreover, we found that fibroblasts and endothelial cells could recruit N1- and N2-TANs via *CSF3*-*CSF3R* and *CXCL2*-*CXCR2* axes, and fibroblasts could promote the adhesion of TANs (primarily N1) via *COL1A1*-*CD44* and *COL1A2*-*CD44* (Fig. 6b and Fig. S20b, c). Particularly, fibroblasts were able to clear N2-TANs by *CXCL12*-*CXCR4*, where *CXCL12* expression was enriched in fibroblasts and *CXCR4* was predominantly expressed in N2-TANs (Figs. 6b, S20d). Additionally, we observed strong colocalization between TANs and other myeloid cells (Fig. 6a), with myeloid cells showing elevated expression of cell adhesion molecules and chemokines (Fig. S20b). Further analysis revealed that macrophages and monocytes can recruit both N1- and N2-TANs (Fig. S20e). These findings suggest that myeloid cells contribute to the recruitment of N1- and N2-TANs, while fibroblasts specifically promote the aggregation and adhesion of N1-TANs, T&NK cells, and other immune cells in the TME, facilitating the clearance of N2-TANs.

Next, we analyzed the maturation and activation mechanisms of the TANs. We found that CD4^+^ T, CD8^+^ T, proliferating T cells (T-pro), and NK cells activated the anti-tumor function of TANs through TNF and IFNG, including cytotoxicity, cytokine production, chemotaxis, migration and IFNγ signaling (Figs. S20f, S21a). Specifically, we found that *IL1B* was involved in cytotoxicity, cytokine production, chemotaxis, and migration of N1-TANs, and it has been recognized as the key gene for N1-TANs to promote cytokine-related gene expression (e.g. *TNF*, *IFNG*) by CD4^+^ T, CD8^+^ T, T-pro cells and NK cells (Fig. S17b). We also investigated whether N1-TANs had any effect on the fibroblasts. These results show that N1-TANs can promote fibroblasts to express substantial chemokines (e.g. *CXCL8* and *CXCL2*) and collagen family genes (e.g. *COL11A1* and *COL1A2*) via *IL1B*-*IL1R1*, indicating the existence of a positive regulatory anti-tumor network based on N1-TANs and fibroblasts (Fig. S21b, c). Additionally, scRNA-seq and ST data were integrated to characterize N1-TANs-fibroblast signaling in the TF region, including fibroblast-facilitated N1-TANs adhesion via *COL1A2* and N1-TANs activated fibroblasts via *IL1B*-*IL1R1* (Fig. 6c). We further directly observed the co-localization of MNDA^+^CD11b^+^ and SLPI^+^CD11b^+^ TANs with COL1A using multiplex IHC (mIHC) on on-treated samples (Figs. 6d, S22a–c). MNDA^+^CD11b^+^ neutrophils were found to be widely distributed in the TF as well as in the vessels, with particular attention to one MNDA^+^CD11b^+^ neutrophil (marked by an asterisk) from the vessel about to enter the TME (Fig. 6d). Together, we characterized the cellular chemotaxis of fibroblasts and discovered that N1-TANs with anti-tumor activity could act on other immune cells and fibroblasts, creating a highly immunogenic TME (Fig. 6e).

### Clinical markers and deep learning models of N1-TANs associated with MTC

Finally, we aimed to construct predictive biomarkers by exploring TANs-associated genes closely correlated with the therapeutic sensitivity and the prognosis of LSCC with MTC. Therefore, we performed a weighted correlation network analysis (WGCNA) on the transcriptomic dataset of TCGA-HNSC, which was clustered into 23 major branches (marked with different colors), among which each branch represented one gene module (Fig. S23a). ssGSEA was applied to analyze the neutrophils infiltration in the TCGA-HNSC cohort by marker genes of Neu_1, Neu_2, Neu_3, and Neu_4 in our scRNA-seq data, including *PDE4B*, *PLEK*, *PROK2*, and *HCAR3* (Neu_1), *MNDA*, *IFITM2*, *CSF3R*, and *FCGR3B* (Neu_2), *S100A12*, *S100A8*, *S100A9*, and *CEBPB* (Neu_3), *SLPI*, *PI3*, *CXCR4*, and *BRI3* (Neu_4) (Fig. S23b). Among all the modules, MEcyan and MEpink showed significantly positive correlations with Neu_1, Neu_2, and Neu_3 (*R* = 0.44, 0.31 and 0.28 in MEcyan, respectively; *R* = 0.53, 0.6 and 0.56 in MEpink, respectively), while relatively weak relevance to Neu_4 (*R* = -0.054 in MEcyan; *R* = 0.3 in MEpink) (Fig. S23b). Then MEcyan and MEpink modules were integrated with neutrophil pseudotime-relative genes (FDR < 0.05), screening 84 and 105 genes, respectively, among which *MX2* and *HCK* were exclusively expressed in N1-TANs of pre-treatment samples compared to all other cells, which was consistent with the pseudotime results (Fig. S24a–d).

**Fig. 7:**
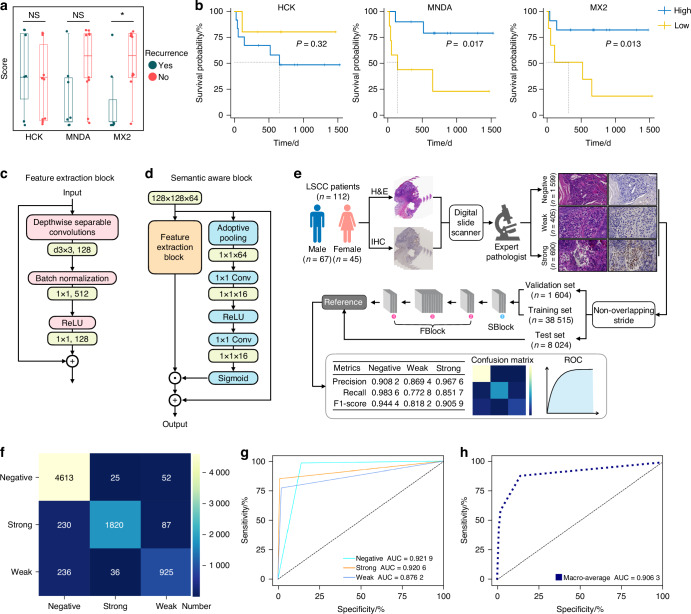
Deep learning models for N1-TANs related makers. **a** Quantitative comparison of HCK, MNDA, and MX2 staining intensity between relapse and non-relapse groups. **b** Recurrence-free survival with different expression levels of HCK, MNDA, and MX2. **c** Flowchart of FBlock. **d** Flowchart of SBlock. **e** Schematic diagram of the deep learning model. **f** Heatmap showing the confusion matrix of the FSNet. **g**, **h** The ROC curves and AUC values of FSNet. A Kaplan-Meier curve was constructed to illustrate overall survival (OS). The mean values across groups were compared using a 2-tailed Student’s *t*-test

Among the 37 LSCC patients treated with MTC in the past 5 years: eight cases had missing paraffin specimens (biopsy at other hospitals), five cases had less than five times of MTC (two were allergic to chemotherapeutic drugs, one did not use chemotherapeutic drugs, and two gave up treatment), and seven cases had 5–10 times of the MTC (obtained positive short-term therapeutic outcomes but gave up treatment for reasons including COVID-19, cost and transportation). The remaining 17 cases had complete treatment and were followed up, of which seven patients had local recurrence and no patient had lymph node metastasis or distant metastasis (Table S5). We divided these 17 cases into two groups based on recurrence and compared their preoperative HCK, MNDA, and MX2 staining scores (Fig. S24e). MX2 expression was significantly higher in the non-recurrence group than in the recurrence group (*P* = 0.036 4) (Fig. 7a). We also divided the patients into high and low groups based on preoperative MNDA, MX2, and HCK staining intensity, and found that the high MNDA and MX2 expression groups were significantly associated with higher survival rates (*P* = 0.017 and *P* = 0.013, respectively) (Fig. 7b).

Furthermore, we attempted to use MX2 in the clinic to allow preoperative biopsy specimens to predict the prognosis of patients treated with MTC. However, IHC staining is expensive and time-consuming, and the quantification of staining intensity varies greatly between pathologists. In contrast, H&E sections are cheaper, more effective, and more stable than IHC. Therefore, we trained and tested a deep-learning model to predict MX2 expression levels in H&E images. First, we built a base layer consisting of a feature extraction block (FBlock) and a semantic aware block (SBlock), named FSNet. FBlock was based on depthwise separable convolutions (DSCs) and inverted bottleneck structure^20,21^ (Fig. 7c). In order to better extract the features of H&E images and improve the recognition ability of the neural network, channel attention was added to the FBlock to form the Sblock^22^ (Fig. 7d). Then, we collected paraffin sections of a total of 112 LSCC patients from our hospital in the last 10 years and performed H&E and IHC staining of MX2. The staining intensity of MX2 was scored by two expert pathologists, finally obtaining a total of 2 694 sections with negative, weakly positive, and strongly positive (*n* = 1 599, *n* = 405 and *n* = 690, respectively). These images were then processed using the stride algorithm for non-overlapping cuts and FSNet (Fig. 7e). The results showed that the FSNet had high recognition and classification ability for HE images with different staining intensities of MX2 in the test set (Fig. 7e, f), and the area under the curve (AUC) values of the receiver operating characteristic (ROC) curve were 0.921 9, 0.876 2, 0.920 6 and 0.906 3 (negative, weak, strong and macro-images, respectively) (Fig. 7g, h), indicating that the model had a high predictive ability of MX2 expression level for HE images.

In summary, our findings suggest that the N1-TANs/fibroblast defense wall forms in response to MTC and effectively combats LSCC.

## Discussion

scRNA-seq and ST were used in tandem to decipher the complex cellular and molecular networks of LSCC in response to MTC treatment. We combined pre- and on-treatment transcriptome data from two patients with LSCC, including 21,108 single-cell levels and four samples at spatial resolution. Fibroblasts accumulated in TF under MTC conditions initiated substantial recruitment and adhesion pathways of N1-TANs, including *CXCL2*-*CXCR2*, *COL1A2*-*CD44*, and so on, while cleared N2-TANs via *CXCL12*-*CXCR4*. Furthermore, mobilized N1-TANs can differentiate into antigen-presenting cells by expressing MHC class I and class II molecular T cells and activate Th17, CD8 T, proliferating Τ, and NK cells in cytokine signaling pathways via IL1B, thereby amplifying anti-tumor immunity. In turn, N1-TANs promote the production of chemokines and collagens by fibroblasts via *IL1B*-*IL1R1*. In this cell-to-cell communication circuit, the MTC acts like a beacon on the Great Wall composed of fibroblasts to mobilize N1-TANs, the sentinels that kill enemy tumors, and expel the dissident N2-TANs, ultimately creating an immunogenic and hypoxia-reversing microenvironment that can destroy cancer cells.

TANs have become essential components of the TME, activating and regulating innate and adaptive immunity.^23^ Furthermore, TANs are derived from PMN and are characterized by diversity and plasticity, with dual potential for anti-tumor and pro-tumor effects.^24,25^ In this study, patients with LSCC had significantly higher levels of TANs with tumor reduction (N1 increased and N2 decreased) after the 5th MTC, which is consistent with the use of TANs as a prognostic marker in colorectal cancer,^26^ gastric cancer^27^ and ovarian cancer.^28^ ST and GO enrichment analyses revealed a heat-induced increment in blood perfusion, vascular permeability, and cellular chemotaxis. This could result in substantial PMN migration from the vessels to the TME and PMN differentiation into N1-TANs by inverting TME hypoxia. The expression of hypoxia-related genes in N1-TANs consistently decreased during the treatment. This supports the hypothesis that hypoxia sets TANs in the pro-tumor state, and hyperoxia activates their anti-tumor potential.^29^ Therefore, the effect of MTC on PMN or immature TANs warrants further investigation.

TANs in LSCC need to be better understood, particularly in MTC. TANs are currently classified into N1 and N2 phenotypes, whereas TANs in the TME have a dynamic spectrum of pro- and anti-tumor states determined by tissue context and multiple external factors.^30^ TANs in LCSS treated with MTC were mostly *MNDA*^+^ N1-phenotypes with fewer *SLPI*^+^ N2-phenotypes. *MNDA*, or myeloid nuclear differentiation antigen, is expressed only in myeloid cells, and its cleavage has been shown to promote neutrophil apoptosis.^31,32^ Conversely, secretory leukocyte protease inhibitor (*SLPI*) promotes neutrophil apoptosis and clearance and inhibits neutrophil elastase (NE).^33^ This may imply that, in the absence of intervention, aged N2-TANs labeled with *CXCR4* would gradually accumulate, promoting the malignant progression of LSCC. Furthermore, IL-1β, a highly regulated inflammatory mediator, produces pro-inflammatory effects when released from the cells by binding to IL-1R1 in the same or nearby cells. IL-1β can effectively promote self-expression and release, amplifying the autoinflammatory response.^34^ Similarly, we discovered that N1-TANs could stimulate fibroblasts to produce chemokines via *IL1B*-*IL1R1*, increasing IL1B expression in N1-TANs. Moreover, IL-1β is also known to be a potent amplifier of adaptive immune responses, activating CD4^+^ T cells, T-pro cells, and NK cells to produce cytokines such as IFNγ,^35^ which is consistent with our findings. In addition, cytotoxicity and degranulation of N1-TANs were significantly enhanced by MTC, which directly killed the cancer cells. It is fascinating to uncover the relationship between the times of MTC treatment and the functional changes in TANs, and deciphering this will help improve our clinical strategies.

TANs can interact with macrophages and T cells and function in the TME.^36–38^ The interaction of TANs with fibroblasts in TME has been reported in hepatocellular carcinoma, where cancer-associated fibroblasts were found to promote the polarization of TANs toward the N2 phenotype via TGF-β.^39^ The interaction of N1-TANs and fibroblasts via IL1B and the decrease in NETs in TF resulted in ECM deposition, which is supported by the fact that NETs can induce ECM remodeling via NE and MMP9.^40^ In contrast to our results, IL1B promotes NETs formation.^41,42^ This disparity could be attributed to the fact that *IL1B*^+^*CXCR4*^-^ N1-TANs were newly derived from PMN and had low levels of ROS (hypoxia being alleviated) and thus not prone to NETosis,^43^ which is consistent with the fact that IL-1β is driven by hypoxia to promote tumor metastasis,^44^ confirming that MTC alters the function of N1-TANs by alleviating their hypoxic status.

Finally, scRNA-seq and ST-identified neutrophil marker genes were combined with TCGA-HNSC transcriptome data to perform WGCNA and ssGSEA analyses, and biomarkers MX2 and HCK of N1-TANs which may be associated with the MTC efficacy in LSCC were identified. The initial biopsies of LSCC treated with MTC were subjected to IHC staining, which revealed a significant positive correlation between MX2 expression and the absence of local recurrence after MTC treatment. Therefore, MX2 can be used as a predictor of MTC in LSCC. We further used deep learning combined with prognostic markers to predict clinical outcomes by feeding raw HE images into our model to obtain MX2 scores, which aided in determining whether patients were initially suitable for MTC treatment. MX2 has not been reported as a prognostic marker for cancer therapy but is associated with promoting neutrophil migration.^45,46^ Here, we combined transcriptomic data, IHC staining results, and a deep-learning model to establish MTC prognostic indicators for LSCC with significant clinical guidance potential. In conclusion, despite the limited sample size of our study, which may not reveal the full LSCC heterogeneity, our findings integrate the strength of scRNA and ST and suggest that MTC could improve the anti-tumor ability of N1-TANs by deciphering the spatial organization and intercellular signatures of the LSCC microenvironment during MTC.

## Materials and methods

### Inclusion criteria and treatment regimens for LSCC patients treated with MTC

Inclusion criteria: (1) The patient presented with a primary lip tumor, which was confirmed by pathologists to be squamous carcinoma; (2) The patient did not exhibit any evidence of pulmonary fibrosis, severe hepatic or renal dysfunction, or other serious systemic disease. (3) All 37 LSCC patients provided written informed consent. Treatment regimens: LSCC patients were treated with MTC twice a week, with an inter-treatment interval of at least 3 days. A standard course consisted of 10 times of MTC treatments. Throughout the course of treatment, blood, liver function, and kidney function tests were conducted to monitor the systemic status of patients. The specific treatment regimen for MTC was as follows: First, the LSCC patient was treated with chemotherapy, which consists of the intravenous administration of 8 mg of pingyangmycin and 10–20 mg of methotrexate (dissolved in 15 mL of saline). Following the intravenous injection of chemotherapeutic drugs over a period of 30 min, microwave thermotherapy (NOVA Company, N-9001) was initiated at a power of 45 watts for a duration of 45 min. During this period, the patient’s lips were maintained at a temperature range of 41 °C to 43 °C by a temperature control system.

### Inclusion criteria for sequencing and collection of clinical samples

Inclusion criteria for sequencing: (1) Primary tumor of the lip; (2) Preoperative biopsy with squamous cell carcinoma diagnosed by pathologists; (3) Patients provided written informed consent to receive MTC treatment at West China Hospital of Stomatology and agreed to undergo re-biopsy if they still had a tumor following half of the treatment course. LSCC tissues were collected before and during MTC after obtaining informed consent from the patients. The study was approved by the West China Hospital of Stomatology Institutional Review Board (WCSHIRB) (No. WCHSIRB-D-2022-324). Fresh tissues were preserved in RPMI-1640 supplemented with 10% fetal bovine serum (FBS) and transported on ice.

### Single-cell sample preparation and sequencing

Single-cell samples were prepared according to the 10× Genomics^®^ Cell Preparation Guide. In brief, clinical samples were collected immediately after surgery, dissociated, minced, and incubated. Suspended cells were washed, counted, and concentrated according to the manufacturer’s instructions. Mixed cell suspensions were analyzed without sorting or enrichment of particular cell types. The cell suspension was then loaded into chromium microfluidic chips with 3’v3 chemistry and barcoded using a 10× Chromium Controller. RNA from the barcoded cells was reverse-transcribed, and sequencing libraries were constructed using Chromium Single Cell 3’ v3 reagent kit reagents. Sequencing was performed using Illumina (NovaSeq) according to the manufacturer’s instructions. To generate a feature barcode matrix, we followed the standard cell ranger (version 4.0.0) workflow to perform alignment, filtering, barcode counting, and UMI counting (https://support.10xgenomics.com/single-cell-gene-expression/software/overview/welcome).

### Visium experiment and sequencing

Fresh tissues were concurrently frozen and embedded in an optically cut tissue compound in liquid nitrogen. An RNA integrity number (RIN) of over seven in tissues was used for spatial gene expression analysis. Cryosections were performed on a Leica CM3050, and bright-field images were taken on a Leica Aperio Versa8 whole-slide scanner at 20× resolution. The Visium Spatial Tissue Optimization Slide & Reagent kit 3’v1 was used to optimize permeabilization conditions for the tissues and construct sequencing libraries according to the Visium Spatial Tissue Optimization User Guide, which was sequenced on the NovaSeq PE150 platform. Raw FASTQ files and histological images were processed using Space Ranger (version 1.2.0) with default parameters. In addition, the filtered gene-spot matrix and fiducial-aligned low-resolution images were used for downstream data analyses.

### Dimension reduction, clustering, and cell annotation of scRNA seq

Potential doublets were detected and removed using DoubletFinder (version 2.0.3; https://github.com/chris-mcginnis-ucsf/DoubletFinder). Cells with high mitochondrial content (≥ 40%) and a low number of features (< 200) were removed. The raw counts were then normalized and scaled using the SCTransform function in Seurat (version 4.2.0),^47^ while the influence of mitochondrial content and cell cycle was regressed using CellCycleScoring. Batch effects were eliminated, and transcriptome data were integrated using canonical correlation analysis (CCA). The top 3 000 variable genes were used for principal component analysis (PCA), followed by FindNeighbors to obtain the nearest neighbors based on 30 PCs, FindCluster to generate 40 cell clusters with a resolution of 2.0, and RunUMAP to visualize cell clusters with the Uniform Manifold Approximation and Projection (UMAP) algorithm. Finally, we combined clustering results and performed differential gene expression analysis (discussed below) between these clusters to compare the top differentially expressed cell-type enriched marker genes previously described in the literature or from the CellMarker 2.0 database to annotate cell clusters.^48^ These marker genes included but were not limited to *KRT7* and *KRT14* for epithelial/cancer cells; *CD3E*, *TRBC2*, and *GNLY* for T&NK cells; *LYZ*, *IL1B*, and *S100A8* for myeloid cells; *IGKC*, *CD79A*, and *IGHG3* for B&Plasma cells; *COL1A1*, *COL1A2*, and *DCN* for fibroblast cells; *SELE*, *CCL21*, and *CFD* for endothelial cells; *TAGLN*, *ADIRF*, and *MYL9* for smooth muscle cells. For further investigation, we sub-clustered immune cells, fibroblasts, endothelial cells, and smooth muscle cells and annotated these cells based on more specific marker genes. Cells expressing double-lineage genes were classified as doublets during annotation and were removed from further investigation. All cellular subsets accounted for at least 5% of the total cells and were distributed in all samples. The FeaturePlot function was used to plot gene expression across cell clusters.

### Spatial transcriptomics data processing

The front of tumor invasion is the area of junction between tumor tissue and normal tissue, located ~250–500 microns on either side of the line of junction between tumor tissue and normal tissue. The gene-spot matrices were imported and analyzed using Seurat. Spots were required to have at least 200 detected genes, and spots with high mitochondrial content (≥40%) were removed. Genes had to have more than ten read counts and express themselves in more than three spots. Spatial transcriptomic data from four slides were processed with SCTransform to normalize the counts, followed by RunPCA, FindNeighbors, and FindCluster to cluster all spots into 14 clusters. SpatialDimPlot visualizes the spatial distribution of spot clusters across slides. AddModuleScore calculated the cell type enrichment scores of spot clusters with the cell type marker gene lists generated by the scRNA-seq data. Finally, the SpatialFeaturePlot function was used to visualize the spatial distribution of the specified cell types. According to the HE staining of the samples, an experienced pathologist defined the epithelial/cancer, fibroblast, and tumor front regions. The image file with outlined regions, feature barcode matrix file, tissue position list file, and scale factor json file were processed by STutility package (version 1.1.1)^49^ to generate epithelial/cancer, fibroblast, and tumor front region as Seurat objects for every slide, respectively. The cell type distribution across these three regions was visualized using SpatialFeaturePlot and multimodal intersection analysis,^12^ which was confirmed by H&E staining. The SpatialFeaturePlot function was used to plot the spatial gene expression across spots.

### Differential expression analysis

Genes differentially expressed between clusters, cell types, and pre- and on-treatment were identified using the FindMarkers function in Seurat with the following parameters: logfc.threshold = 0.25, test.use = “wilcox”, min.pct = 0.1, and adjusted *p*-values were calculated based on the Bonferroni correction. We used GSE101584 gene expression data to identify N1-TAN and N2-TAN marker genes.^50^ After data cleaning, removal of batch effects, and probe annotation, the limma package (version 3.52.1)^51^ was used to identify DEGs between N1-TANs and N2-TANs. Genes with |log2-fold change | >1 and FDR < 0.05 were considered N1-TANs and N2-TANs marker genes. Dot plots, heatmaps, and volcano plots of gene expression across different cell types and conditions were generated using the scCustomize (version 0.7.0, https://github.com/samuel-marsh/scCustomize), ComplexHeatmap (version 2.12.1)^52^ and ggplot2 (versions 3.4.0, https://ggplot2.tidyverse.org) packages, respectively.

### Functional enrichment analysis

GO enrichment analysis was performed on differentially expressed genes (DEGs) with log2-fold change >1 and FDR < 0.05, using the enrichGO function in the clusterProfiler package (version 4.4.4)^53^ with default parameters. The ggplot2 package generated dot and bar plots of the enrichment analysis results. Relevant functional gene lists were downloaded and processed using the msigdbr (version 7.5.1, https://CRAN.R-project.org/package =msigdbr) and KEGGREST packages (version 1.36.3, https://www.bioconductor.org/packages/release/bioc/htmL/KEGGREST. html), as previously described. Functional enrichment scores for single cells or spots were calculated using AddModuleScore, which was then visualized using ggplot2, and the *p*-values for comparisons were calculated using the ggsignif package (version 0.6.3, https://const-ae.github.io/ggsignif/). Gene set enrichment analysis of the hypoxia pathway between pre-treatment and on-treatment N1- and N2-TANs was performed using clusterProfiler, which was then visualized using the enrichplot package (version 1.16.2, https://github.com/YuLab-SMU/enrichplot).

### Trajectory reconstruction and RNA velocity estimation

The pseudotime trajectory of CD4^+^ T cells, CD8^+^ T cells, and neutrophils was reconstructed using Monocle3 (version 1.3.1, https://cole-trapnell-lab.github.io/monocle3/), following the manufacturer’s instructions. Genes that changed as a function of pseudotime were identified using the graph_test function. A ComplexHeatmap was used to generate a gene expression heatmap along the pseudotime. To validate the putative differentiation trajectories generated by Monocle3, we performed RNA velocity estimation based on the spliced and unspliced transcript data of scRNA-seq loom files with the velocity. R package (version 0.6, https://github.com/velocyto-team/velocyto.R) and RNA velocity vectors were visualized on UMAP by Gaussian smoothing on a regular grid. Furthermore, we visualized the probability density of these two conditions’ cells along the pseudotime trajectory to interpret the differences between pre-treatment and on-treatment at the trajectory, cell population, and gene expression levels. Finally, we compared the smooth curves of gene expression and functional scores of pre-treatment and on-treatment samples inspired by Hector Roux de Bézieux (https://hectorrdb.github.io/condiments/articles/condiments.html).

### Survival analysis

Gene expression matrix and patients’ clinical data from TCGA-HNSC were accessed and processed using TCGAbiolinks (version 2.25.3).^54^ The cell infiltration score of each sample was calculated using the ssGSEA function in the GSVA package (version 1.44.5)^55^ based on the cell-type marker identified in the scRNA-seq. Further survival analysis was performed using survival (version 3.3–1, https://CRAN.R-project.org/package =survival) and survminer packages (version 0.4.9, https://CRAN.R-project.org/package =survminer). Potential cutting points were yielded with maxstat.test function from the maxstat package (version 0.7–25, https://CRAN.R-project.org/package =maxstat) using the maximally selected rank statistics. The patients were divided into two groups based on their cutoff points. Finally, a Kaplan-Meier survival curve was constructed using the survival function. The two-sided log-rank test was used to compare the Kaplan-Meier survival curves.

### Cell-cell communication analysis

The ligand-receptor interaction between cell clusters was inferred by CellChat (versions 1.6.0),^56^ which could provide more consolidated and predictable interactions. We first extracted normalized gene expression data and annotation data of cell clusters to create a CellChat object, followed by identifying overexpressed ligands, receptors, and their interactions, which were then projected onto the protein-protein interaction (PPI) network to smooth gene expression values. Ligand-receptor interactions were filtered if the number of expressed cells in one group was <25%, and the minimum number of cells in each cell group for cell-cell communication was set to 10. Next, we used NicheNet (version 1.1.1)^57^ to identify the downstream genes regulated by cell-cell communication. Genes expressed in >10% of the “sender” or “receiver” cells were considered. The top 20 ligands based on ligand activity and the top 250 target genes based on potential regulatory scores were screened. The regulatory score and gene expression were visualized as a heatmap or dot plot using the gglpot2.

### Immunohistochemistry (IHC)

Surgically excised specimens were obtained from West China Stomatological Hospital, Sichuan, China. After fixation in 10% neutral formalin for 24–48 h, the specimens underwent routine dehydration, transparency, embedding, and sectioning for subsequent immunohistochemical staining. Four-micrometer-thick paraffin-embedded sections were deparaffinized using xylene, followed by dehydration through a series of graded ethanol. Subsequently, the slides underwent antigen retrieval by incubating them in a 1 mM EDTA buffer (pH 8.0) at 120 °C for 20 min. To block endogenous peroxidase activity, the specimens were treated with a 3% hydrogen peroxide solution at room temperature for 10 min. Following that, the sections were incubated with primary antibodies targeting specific antigens: CD11B (66519-1-lg, Proteintech, Wuhan, China), SLPI (bs-6849R, Bioss, Beijing, China), MX2 (bs-10985R, Bioss, Beijing, China), MNDA (bs-17692R, Bioss, Beijing, China), and HCK (bs-1438P, Bioss, Beijing, China), at 4°C for 12–18 h. The antigen-antibody complexes were visualized using either the UltraSensitive SP IHC Kit (KIT-9710, Maixin Biotech, Fuzhou, China) or the DoubleStain IHC Kit: DouSP IHC Kit (KIT-9999, Maixin Biotech, Fuzhou, China) to simultaneously assess the levels of two different antigens from the same tissue in immunohistochemical staining.

### Multiplex immunohistochemistry (OPAL) staining protocol, image acquisition, and data analysis

Multiplexed IHC was stained with opal reagent (TSA: Tyramide signal amplification). Consecutive staining was performed by heat-induced antigen retrieval followed by incubation with primary antibody. The signal was amplified and detected with Opal polymer horseradish peroxidase and dye (Akoya Biosciences). The sections were then subjected to heat-induced antibody stripping and incubated with the next antibody until the last and spectral DAPI. All Opal reagents were used at a dilution of 1/100. Slides were scanned with a PhenoImager Fusion (Akoya Biosciences) to split the spectra from different fluorescence channels. We use a multiplex phenotyping module to classify different cell phenotypes and the marker co-expression. Zhi wei biological technology (zhengzhou) Co., was entrusted to run the whole workflow.

### Weighted gene co-expression network analysis (WGCNA)

The WGCNA package (version 1.71)^58^ was used to construct the weighted gene co-expression network using the gene expression matrix from TCGA-HNSC as input and a standard WGCNA protocol to determine the regulatory gene network behind N1-TANs infiltration into HNSCC tumors. First, the soft-thresholding power was visualized and selected using the pickSoftThreshold function. Next, the most negligible power with a scale-free topology fit index above 0.80 was chosen, which was 4 in our study. We then transformed adjacencies into a topological overlap matrix, calculated dissimilarity, and produced a dendrogram of genes using hierarchical clustering, producing 23 gene modules. Eigengenes of each module were then calculated, and correlation analysis between the eigengenes of each module and the ssGSEA infiltration score of neutrophil subclusters was performed and visualized using the labeled heatmap function.

### Dataset for deep learning

The 112 H&E-stained and 112 IHC-stained MX2 LSCC tissue sections were analyzed and scored by two pathologists (both had at least 7 years of clinical experience), and patches of inflammatory cell-enriched areas were extracted at ×200 magnification. The slides were scanned and preserved with a KF-PRO-005-EX scanner (KFBIO, Ningbo, China). Then this dataset was extended by stride of 256 × 256 pixels without overlapping to obtain a total of 48 143 patches, with training set (*n* = 38 515), validation set (*n* = 1 604), and test set (*n* = 8 024).

### Deep learning network setting

The deep learning network was implemented by PyTorch. We used the Aadm optimizer with an initial learning rate of 1e^-4^ and set β1 to 0.9, β2 to 0.999, and ϵ to 1e^-8^, respectively. Learning rate drops 0.1 every 40 epochs. We trained the network from scratch with a batch size of 32 and 100 epochs. The whole training was conducted on one NVIDIA 3090 GPU.

## Supplementary information

Revised Supplementary information

## Data Availability

We have organized all the raw sequencing data and are prepared to upload it to the GEO database.
