# Supplementary material for: Single-cell and spatial transcriptomics reveals an anti-tumor neutrophil subgroup in microwave thermochemotherapy-treated lip cancer
Source: Int J Oral Sci. 2025 May 13;17:40. doi: 10.1038/s41368-025-00366-8 (PMC12075663; doi:10.1038/s41368-025-00366-8)
Supplement: Supplementary file 1 — Revised Supplementary information [file 41368_2025_366_MOESM1_ESM.docx]

Supporting Information

**Single-cell and spatial transcriptomics reveals an anti-tumor neutrophil subgroup in microwave thermochemotherapy-treated lip cancer**

**Bing-jun Chen^1†^, Hua-yang Fan^1†^, Xin Pang^1^, Ze-liang Shen^2^, Rui Gao^3^, Hao-fan Wang^1^, Zhen-wei Yu^1^, Tian-jiao Li^1^, Mao Li^2^, Ya-ling Tang^2*^, Xin-hua Liang^1^**

**
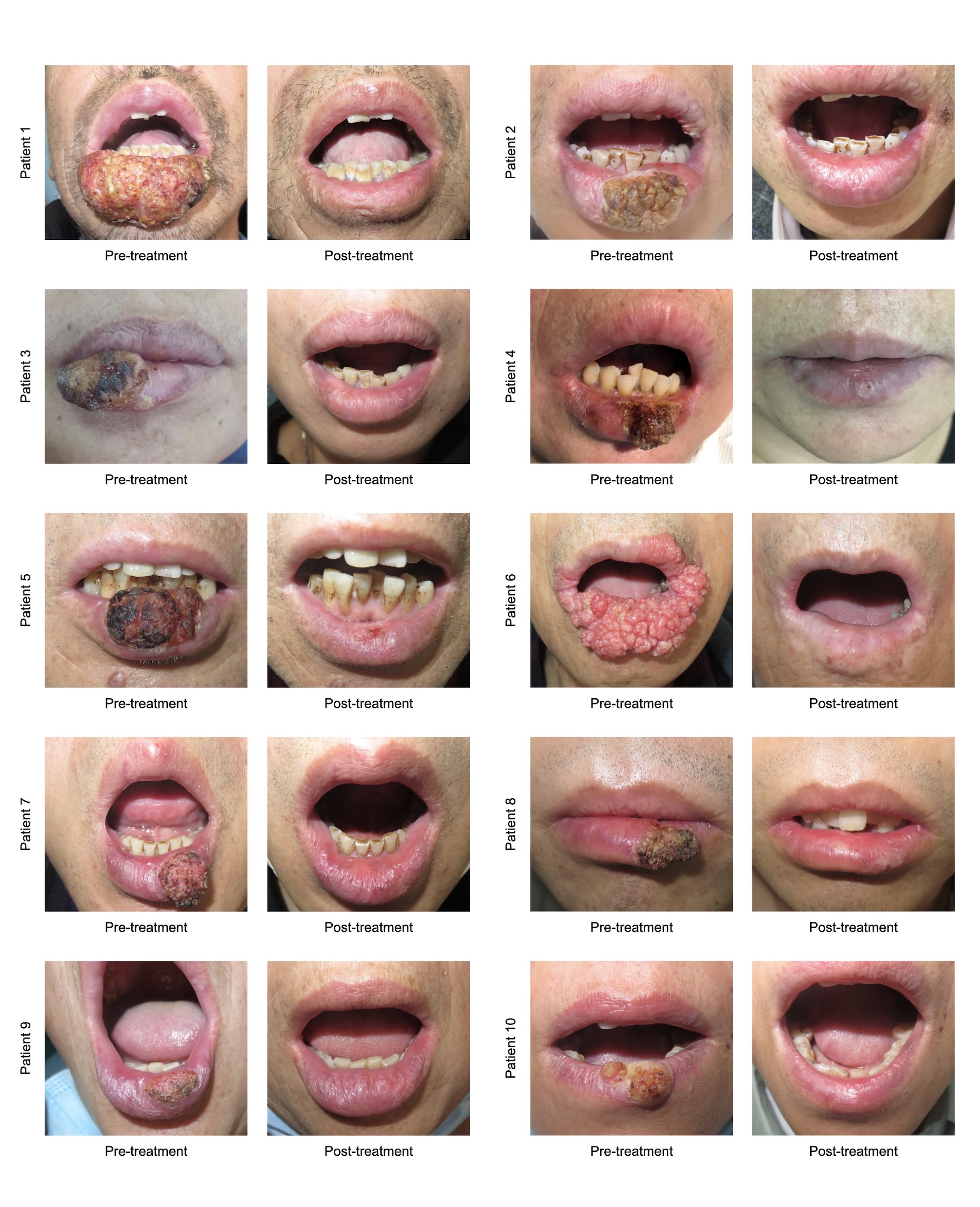
**

**Fig. S1** Cases of MTC treatment for LSCC, related to Fig. 1. Figures showing 10 patients with LSCC who achieved satisfactory MTC outcomes, including pre-treatment and follow-up photos.


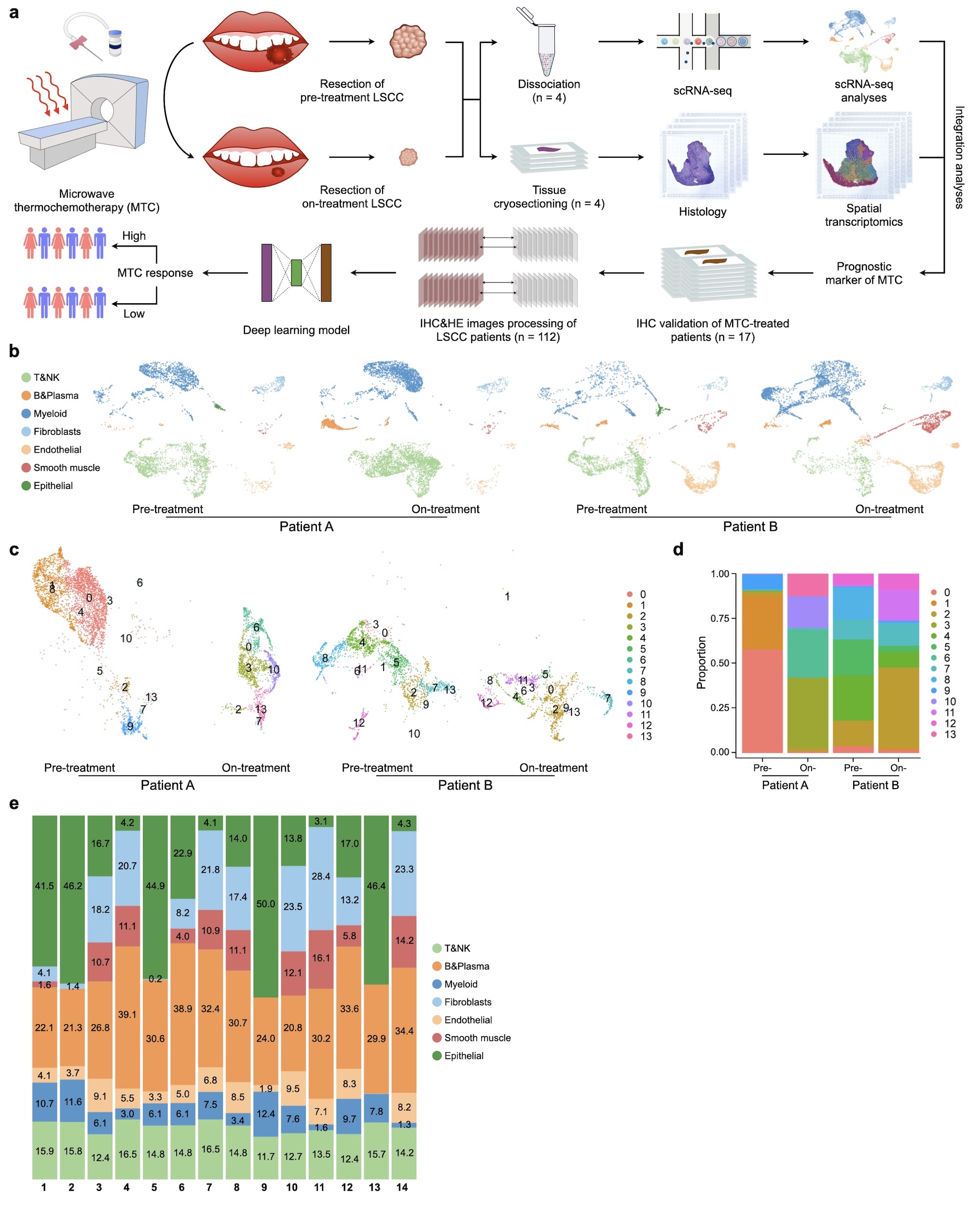


**Fig. S2** Single-cell and spatial transcriptomics atlas of LSCC, related to Fig. 1. **a** Schematic of the experimental design and analysis. **b** UMAPs of 4 samples of patients A and B, showing 7 clusters in each plot. **c** UMAPs of 4 samples based on ST, showing 14 spatial point clusters. **d** Bar plots showing proportions of 14 spatial point clusters in each sample based on the ST data. **e** Bar plots showing tissue distribution of 7 major cell types, with numbers indicating the percentage of cell subtypes in spatial clusters.

**
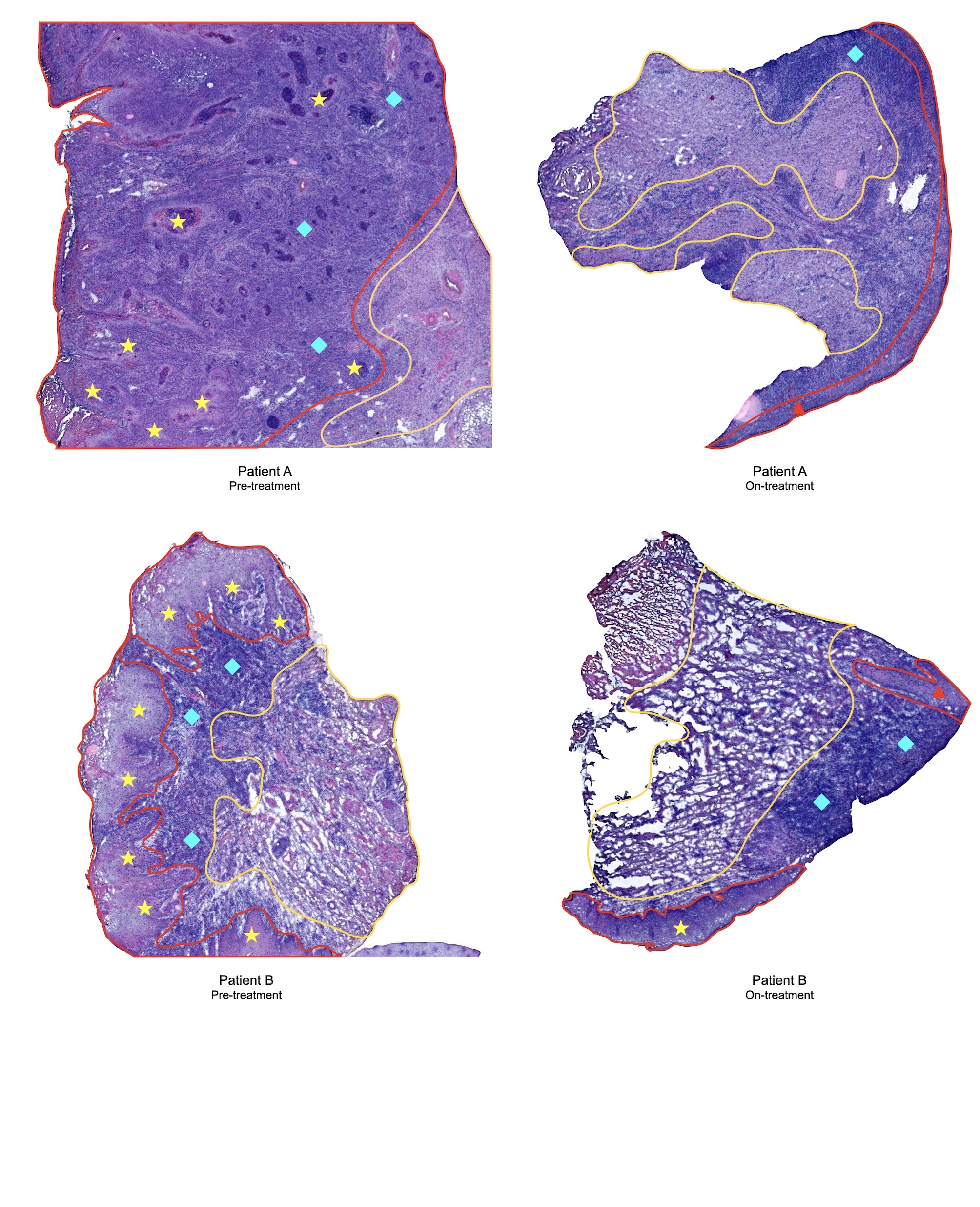
**

**Fig. S3** H&E plots of LSCC, related to Fig. 1. H&E plots of the four samples are presented with the tumor region outlined in red and the stromal region outlined in yellow. Yellow pentagrams indicate typical tumor areas, red triangles denote normal epithelium, and blue diamonds highlight areas of immune cell infiltration.

**
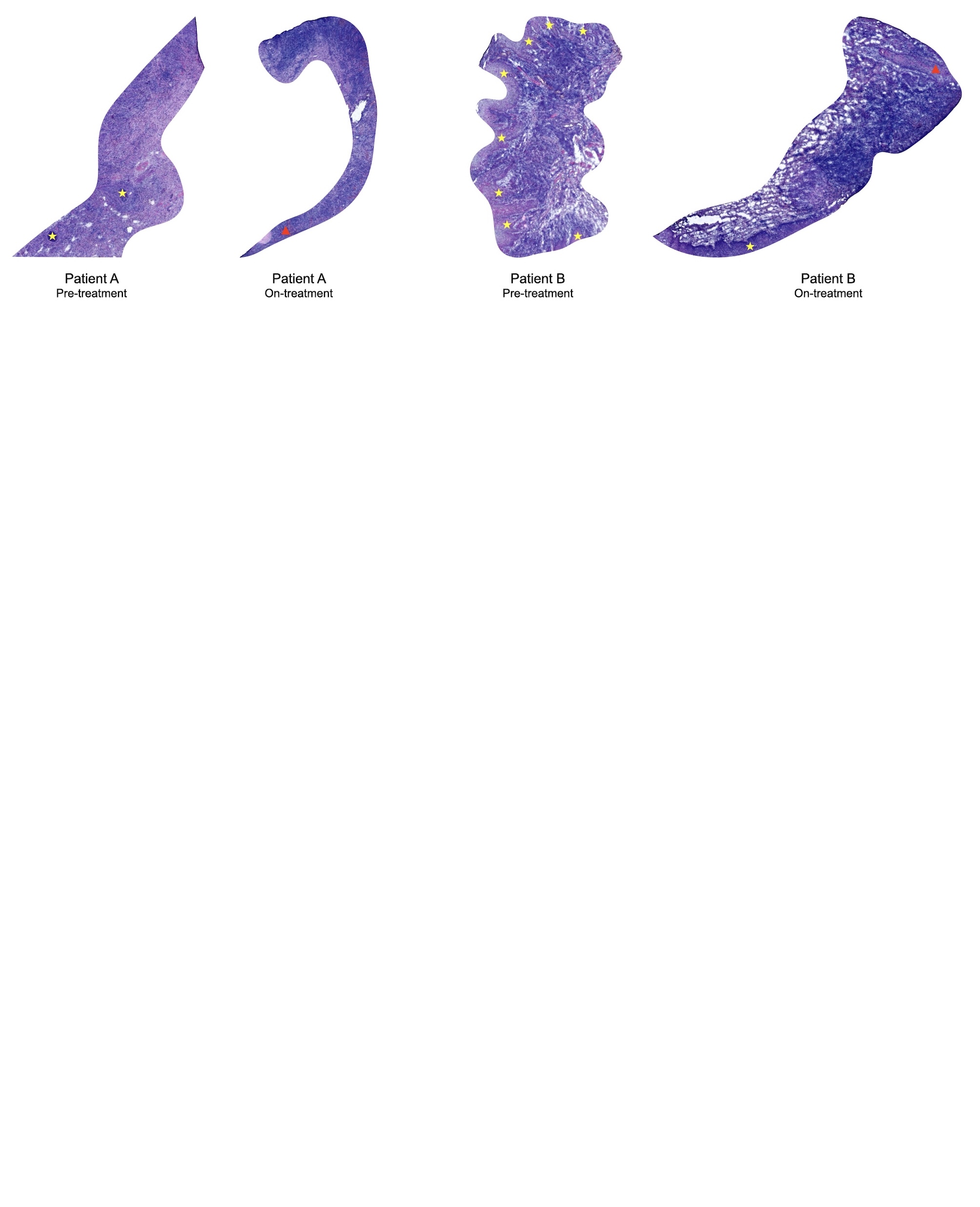
**

**Fig. S4** H&E plots of LSCC, related to Fig. 2. Typical tumor areas in the TF region are marked by yellow pentagons, while normal epithelium is indicated by red triangles.


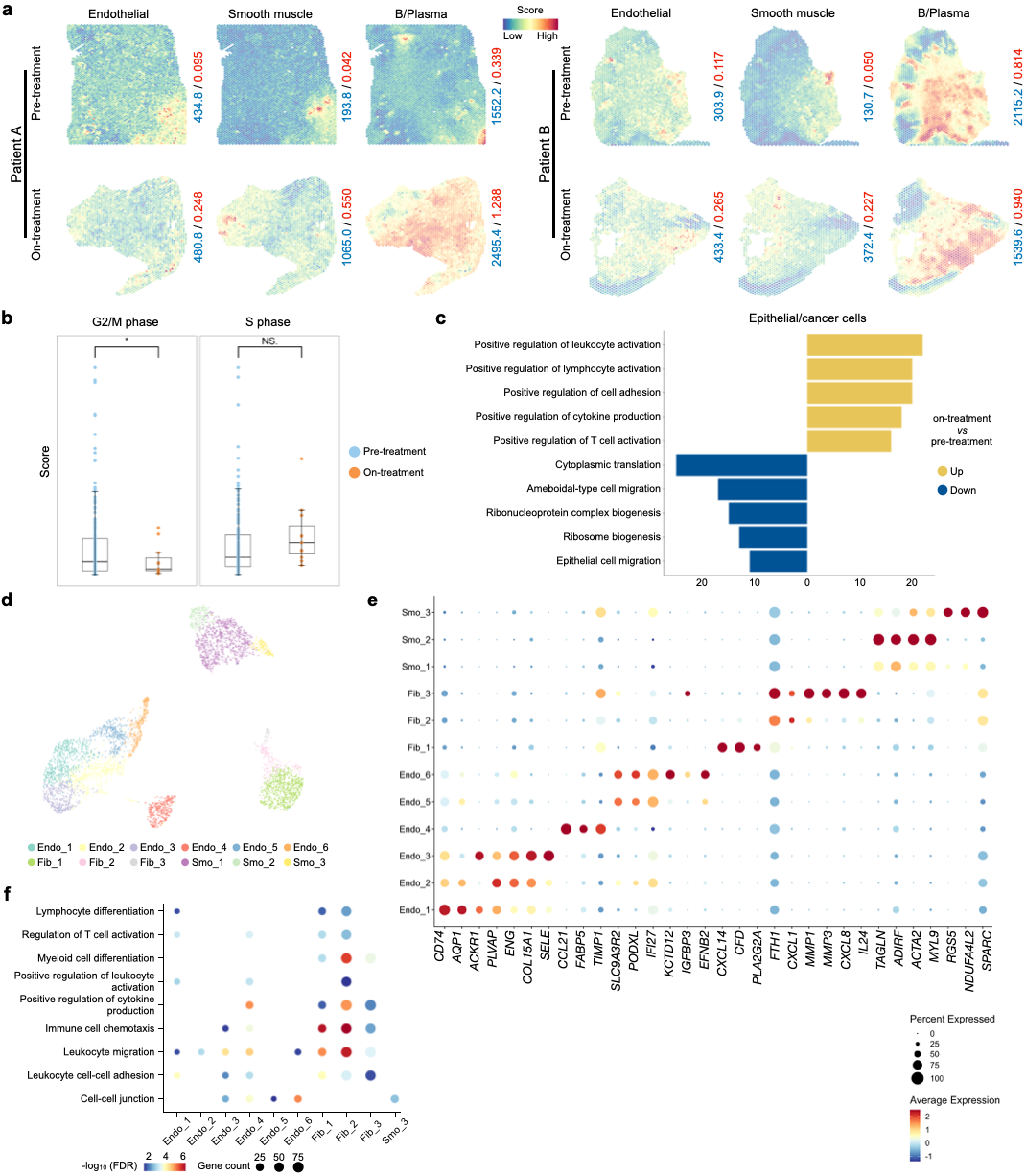


**Fig. S5** The heterogeneity of TME and the functional analysis of epithelial/cancer cells, related to Fig. 2. **a** Spatial distribution of endothelial cells, smooth muscle cells and B&Plasma cells. The blue numbers on the right side of the ST plot indicate the total infiltration score for each cell type throughout the image, and the red numbers indicate the mean score of every spot. **b** Box plots showing the G2/M and S phase scores of epithelial/cancer cells in TF of pre- and on-treatment. **c** Bar plots showing GO enrichment analysis of epithelial/cancer cells (on-treatment vs pre-treatment). **d** UMAP of interstitial cells from patients A and B. **e** Dot plot showing average expression of known markers in indicated cell clusters. **f** Dot plot showing GO enrichment analysis of endothelial, fibroblast, and smooth muscle cell subpopulations. The mean values across groups were compared using a 2-tailed Student’s t-test.


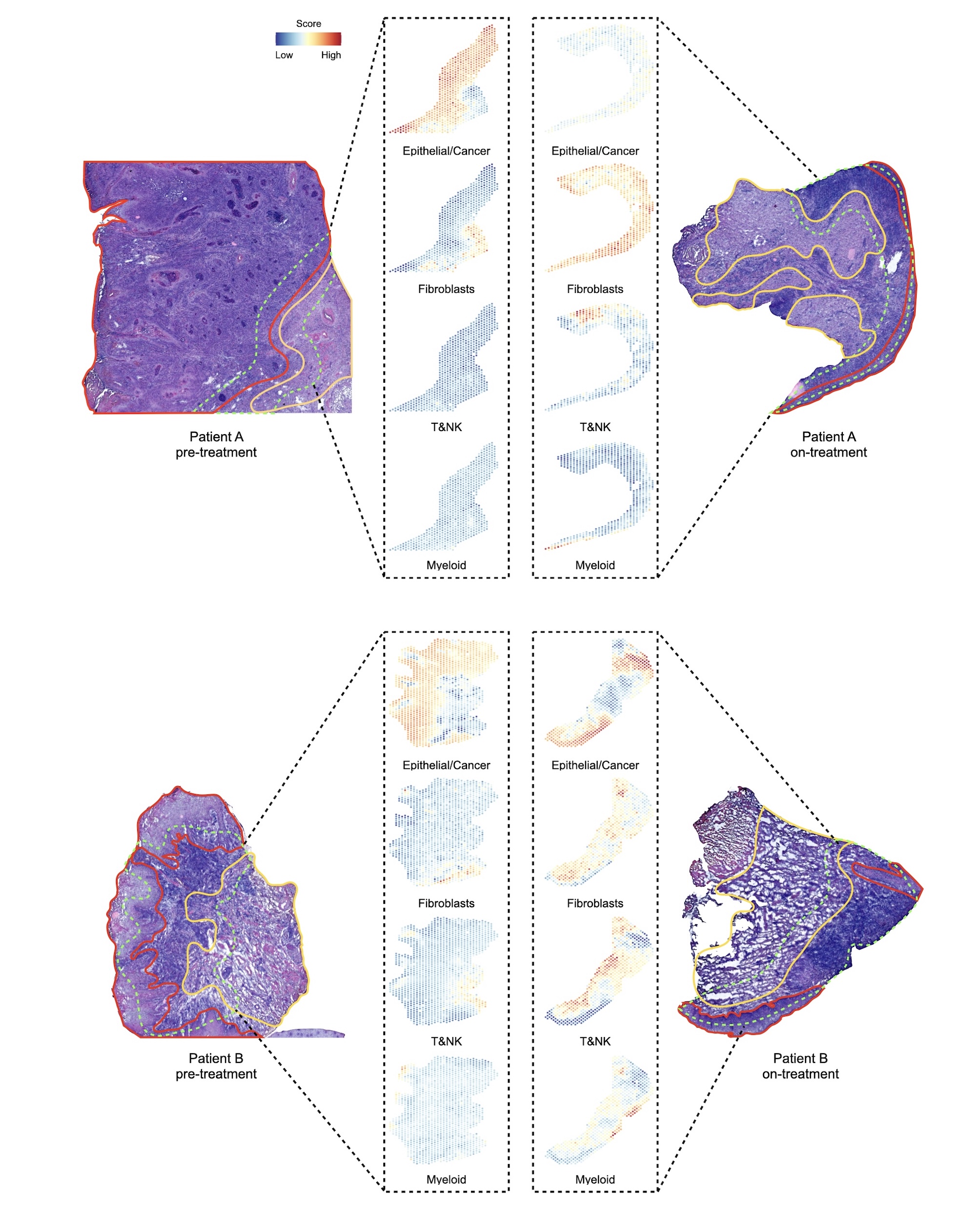


**Fig. S6** Cellular distribution in TF, related to Fig. 2. Spatial distribution of epithelial cells, fibroblasts, T/NK cells and myeloid cells in the tumor front.

**
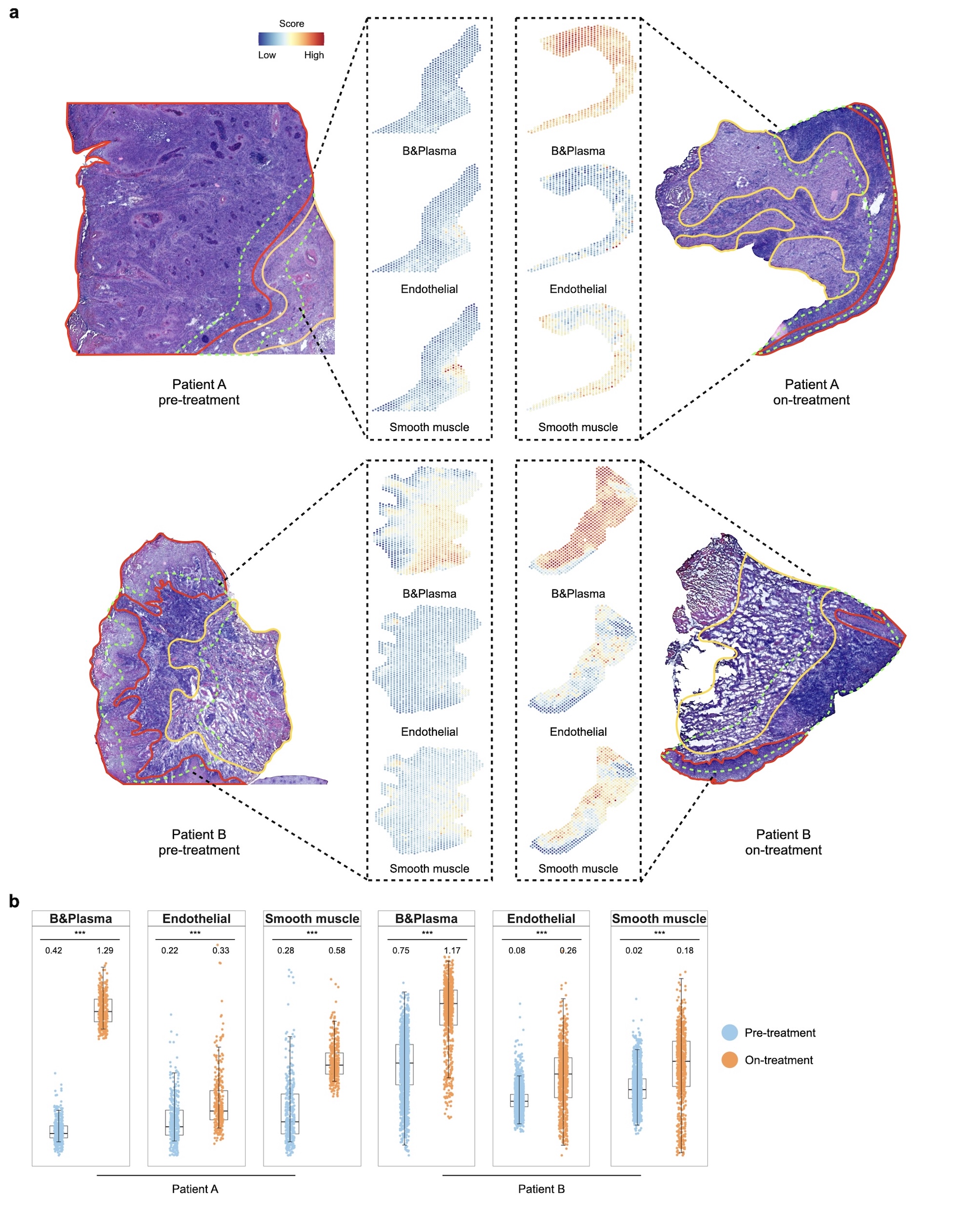
**

**Fig. S7** Cellular distribution in TF, related to Fig. 2. **a** Spatial distribution of B/Plasma cells, endothelial, and smooth muscle cells in the tumor front. **b** Box plots showing the infiltration scores of different cell types in TF of pre- and on-treatment, with specific scores labeled.


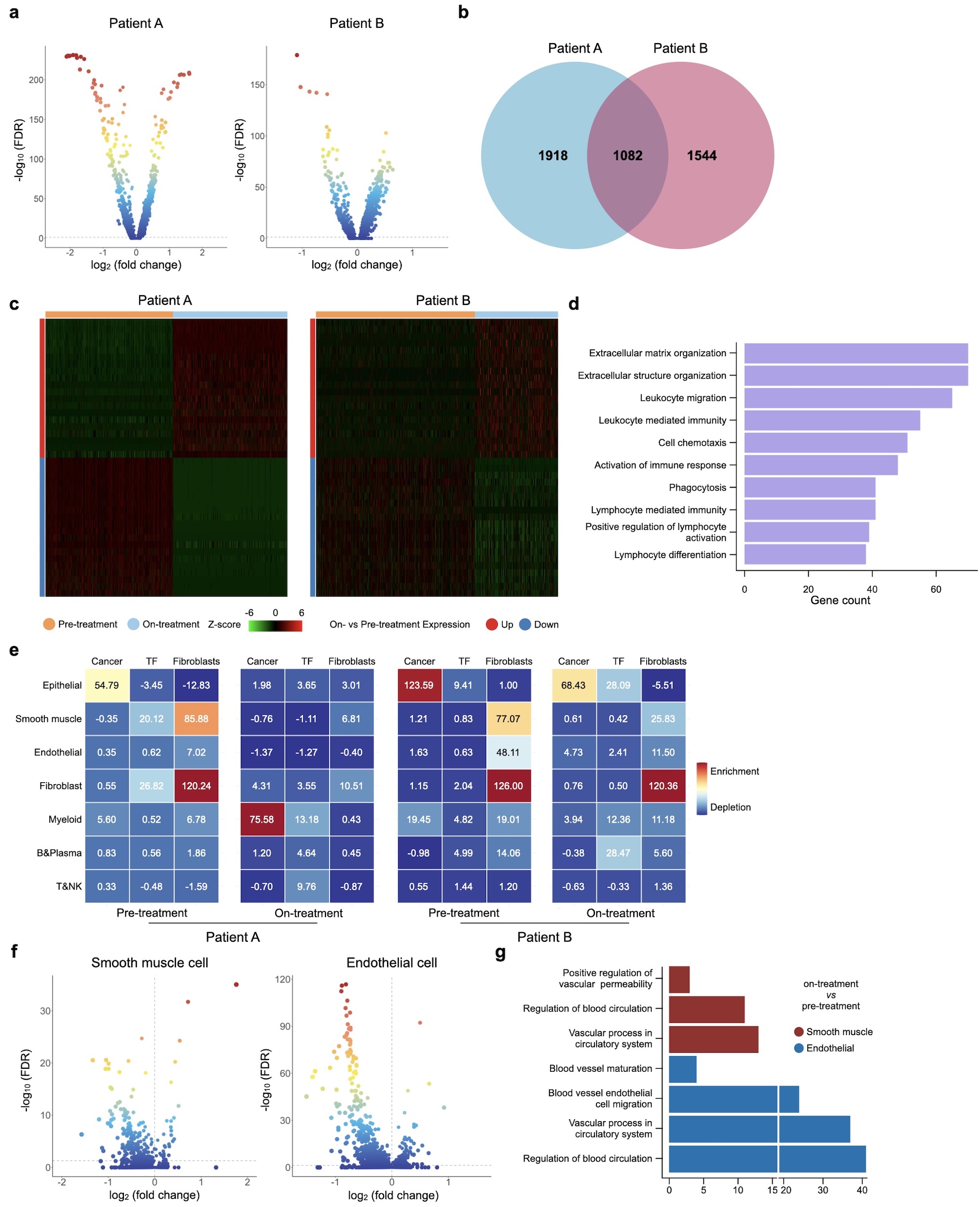


**Fig. S8** DEGs of TF and the heterogeneity of TME, related to Fig. 2. **a** Volcano plot of DEGs in TF regions. **b** Venn diagram of overexpressed DEGs in TF regions of patients A and B. **c** Heat map of DEGs in TF regions. **d** Bar plots showing GO enrichment analysis of upregulated genes in on-treatment TF spots. **e** The MIA heatmap of all scRNA-seq-identified cell types and ST-defined regions, with their specific correlation scores labeled in the corresponding boxes. **f** Volcano plot of DEGs in smooth muscle cells and endothelial cells in the TF regions (on-treatment *vs* pre-treatment). **g** Bar plot showing GO enrichment analysis of smooth muscle cells and endothelial cells (on-treatment *vs* pre-treatment).

**
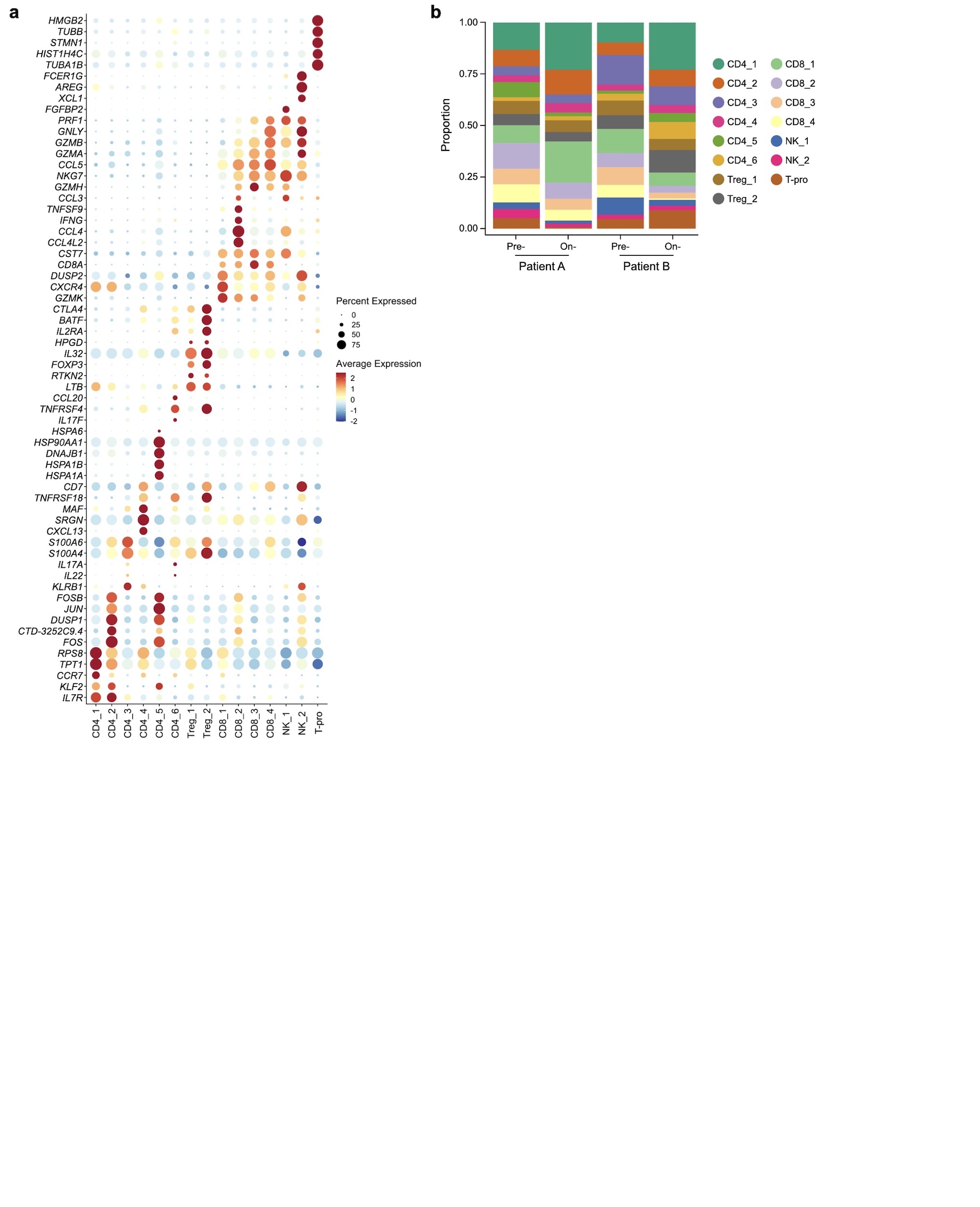
**

**Fig. S9** Sub-clustering of T&NK cells in LSCC, related to Fig. 3. **a** Dot plot showing average expression of known markers in indicated cell clusters. **b** Bar plots showing proportions different T&NK cell subsets in each sample.

**
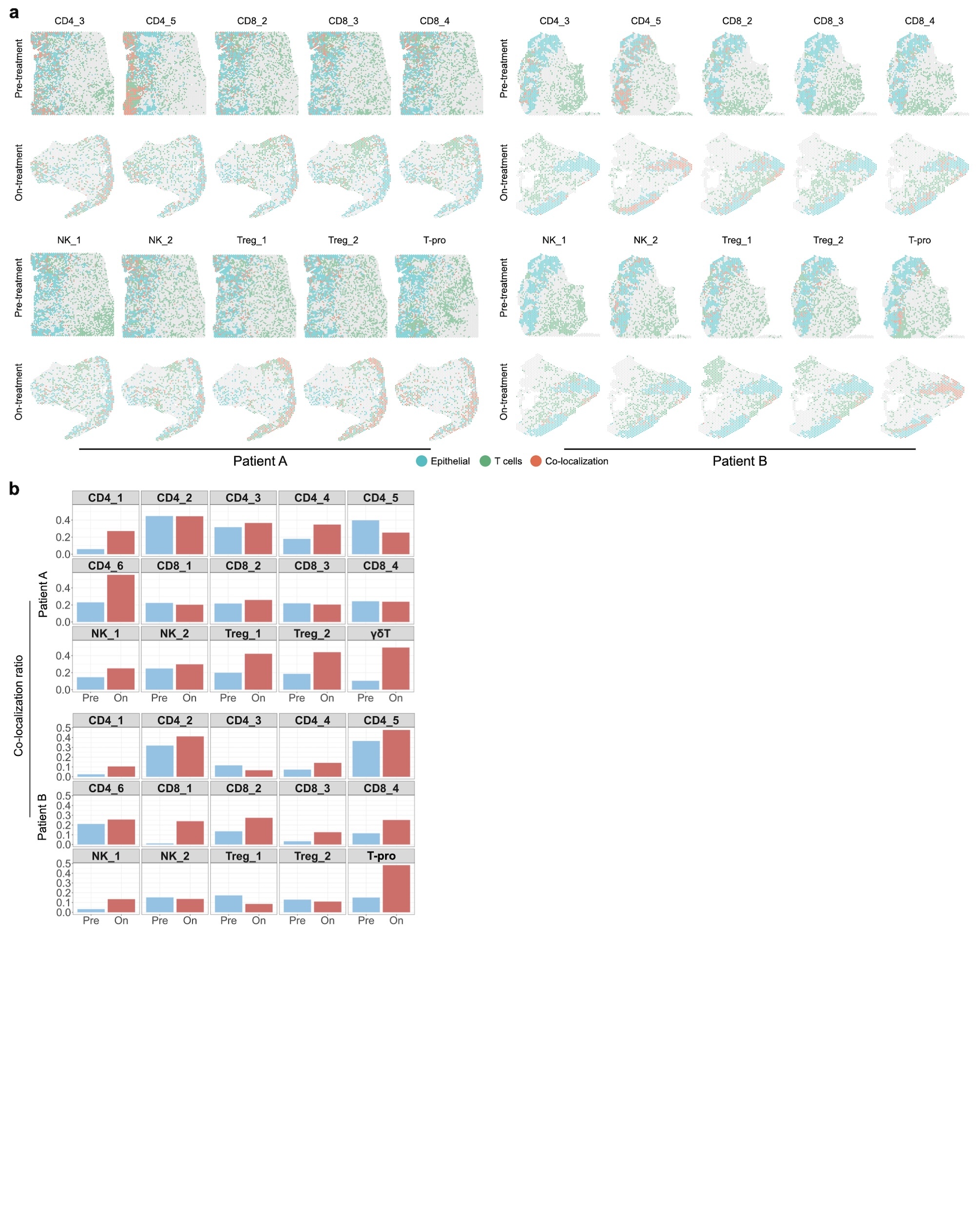
**

**Fig. S10** Spatial distribution of T&NK cells in LSCC, related to Fig. 3. **a** and **b** Spatial colocalization and quantitative statistical histogram of T&NK cells and epithelial/cancer cells

**
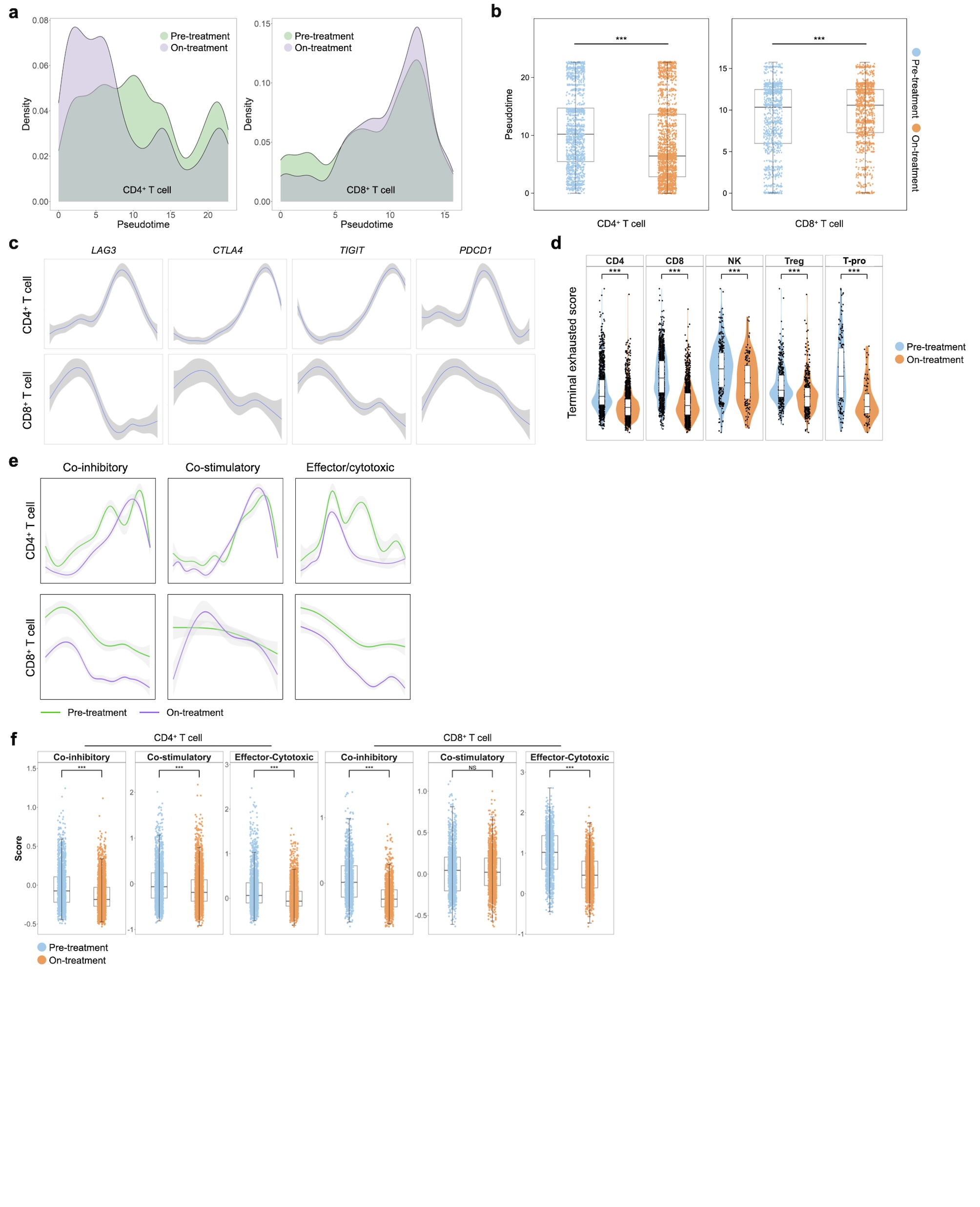
**

**Fig. S11** Pseudotime and function analysis of T&NK cells, related to Fig. 3. **a** The probability density plot of CD4^+^ and CD8^+^ cells along the pseudotime trajectory. **b** Histogram of the difference between the pseudotime of CD4^+^ and CD8^+^ T cells. **c** Smooth curves showing co-inhibitory genes expression along the CD4^+^ and CD8^+^ trajectory. **d** Violin plots of terminal exhausted scores of T&NK cells. **e** Smooth curves showing the score of co-inhibitory, co-stimulatory, and effector/cytotoxic of CD4^+^ and CD8^+^ cells in pre- and on-treatment. **f** Score of co-inhibitory, co-stimulatory, and effector/cytotoxic of CD4^+^ and CD8^+^ cells in pre- and on-treatment.


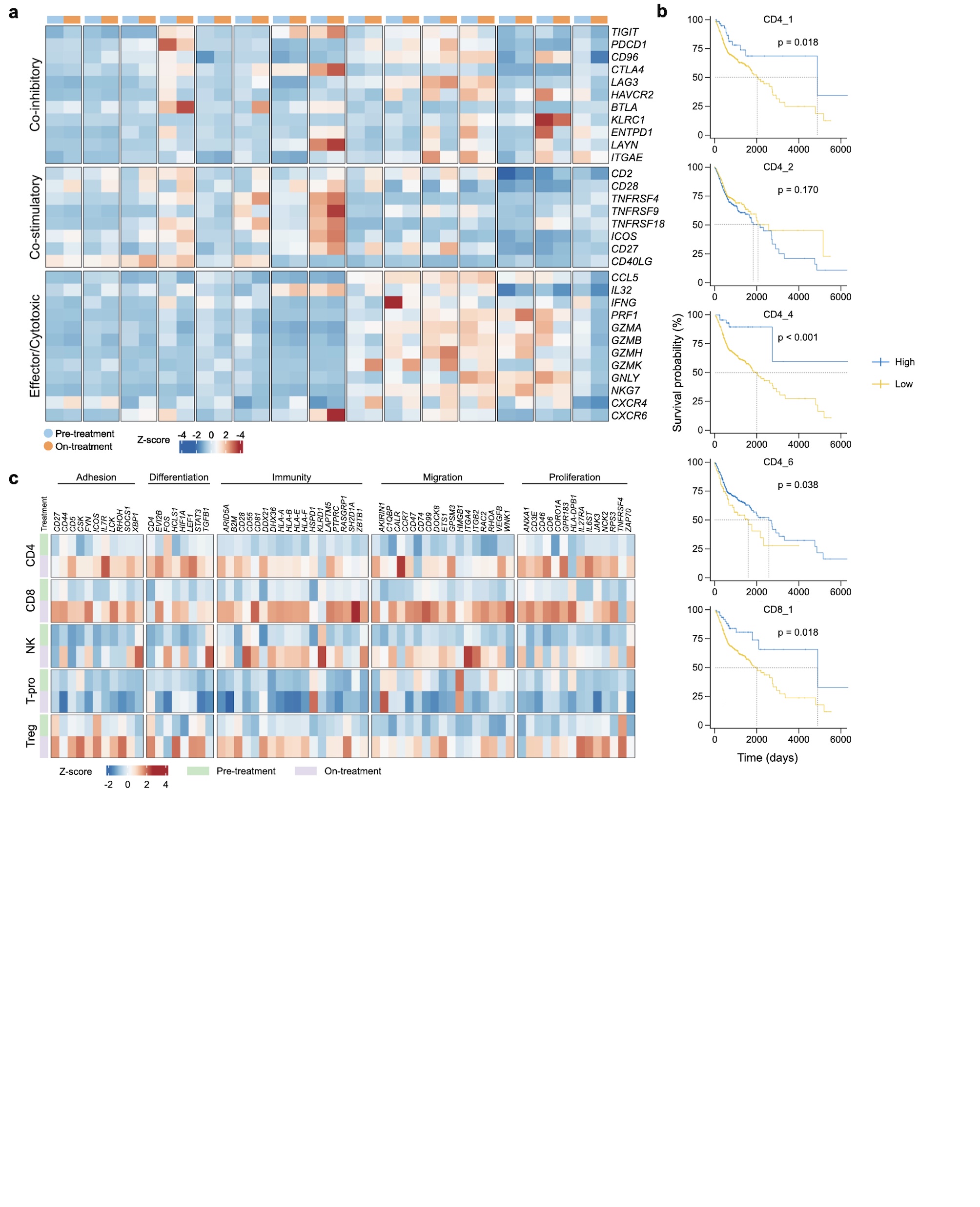


**Fig. S12** Pseudotime and function analysis of T&NK cells, related to Fig. 3. **a** Heatmap showing Z-scored expression of co-inhibitory, co-stimulatory and effector/cytotoxic function associated genes across T&NK subsets. **b** Overall survival of T cell subpopulations infiltration in TCGA HNSC cohort. A Kaplan-Meier curve was constructed to illustrate overall survival (OS). **c** Heatmap showing the expression levels of T&NK genes that exert different effects on myeloid cells. The mean values across groups were compared using a 2-tailed Student’s t-test.


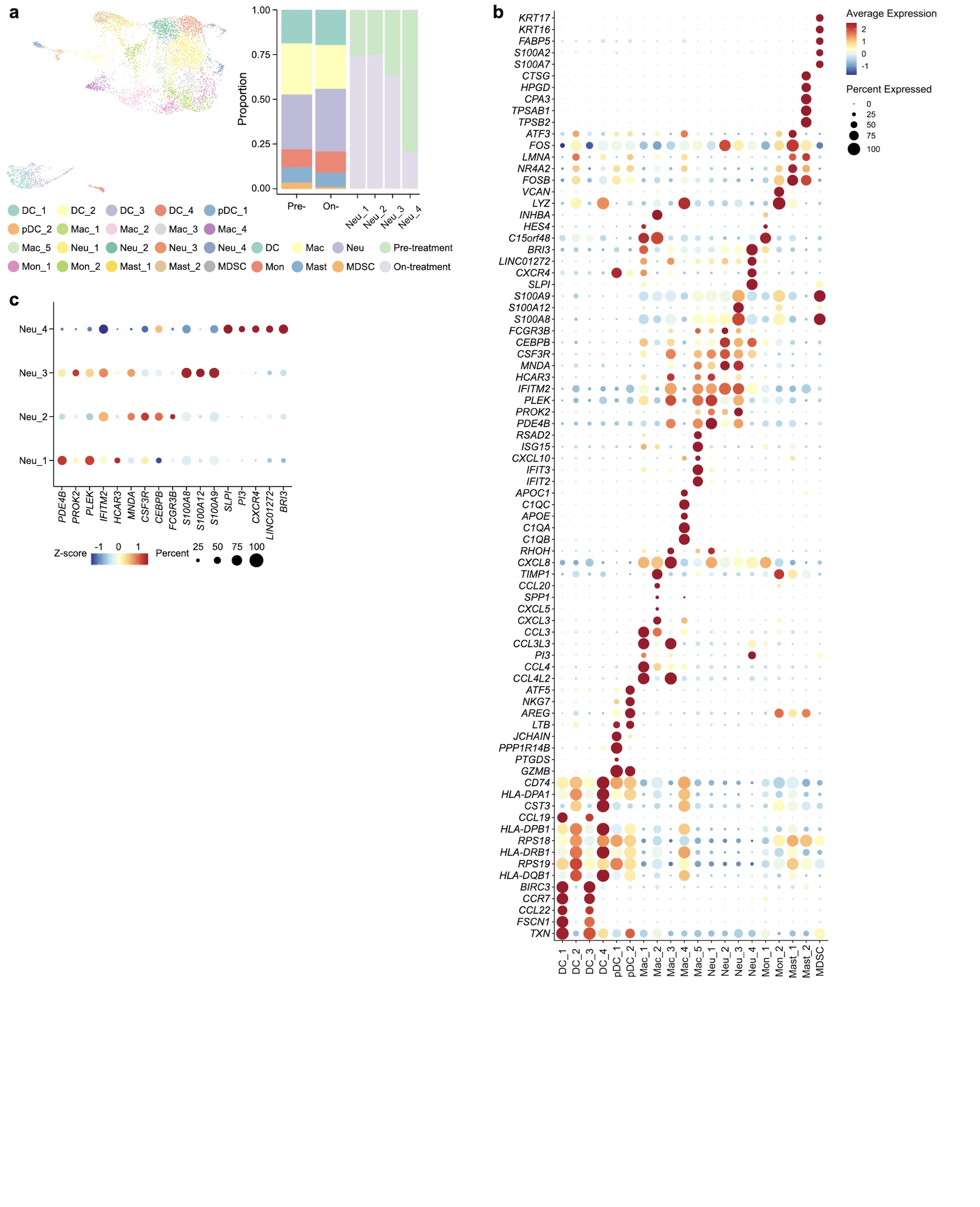


Fig. S13 Neutrophil characterization, related to Fig. 4. a UMAP of myeloid cells from patients A and B, and bar plots showing proportions of myeloid subpopulations and Neu subsets in pre- and on-treatment. b Dot plot showing average expression of known markers in indicated cell clusters. c Dot plot showing average expression of known markers in indicated cell clusters.


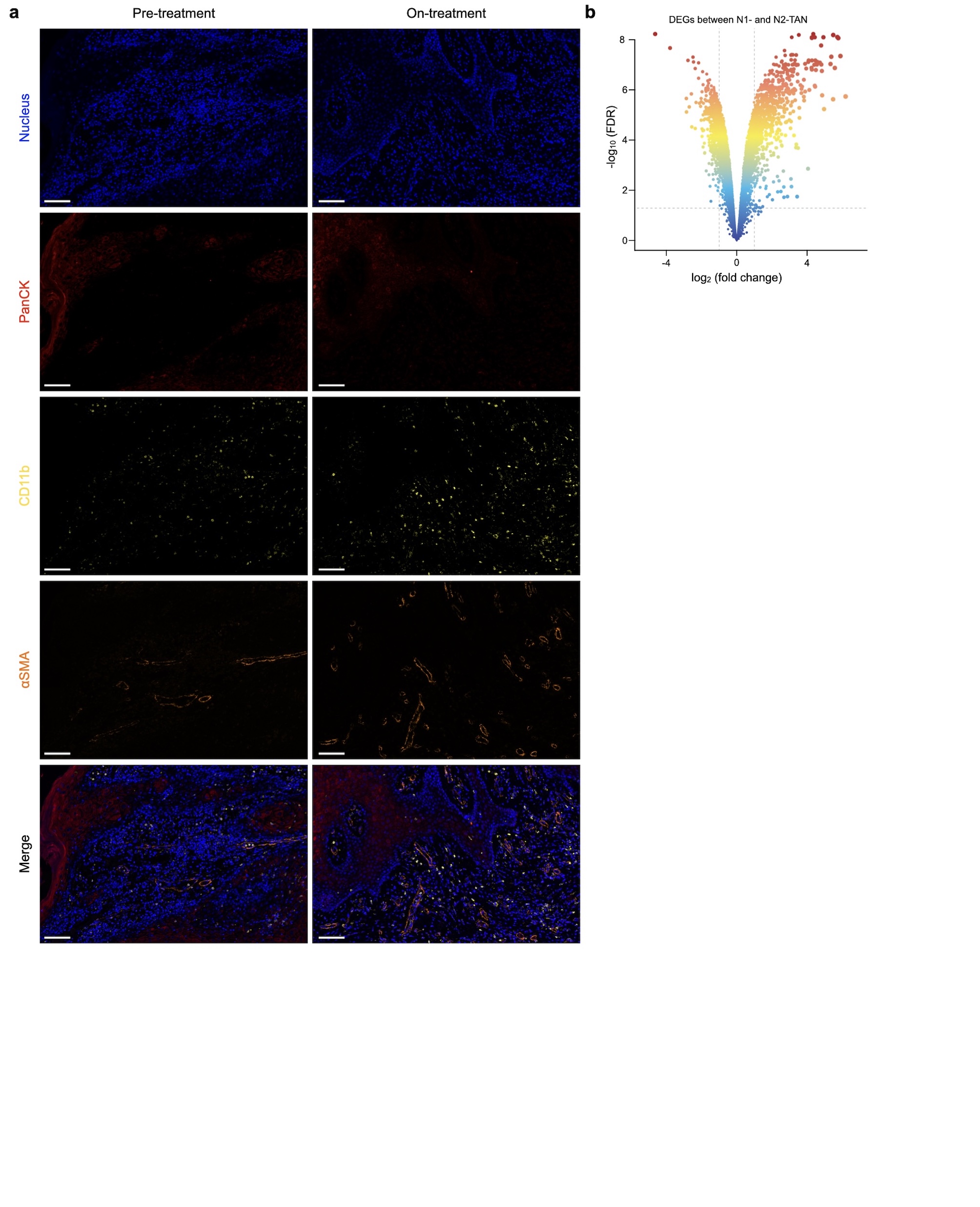


Fig. S14 Neutrophil characterization, related to Fig. 4. a IF staining of nucleus, PanCK, αSMA, and CD11b in LSCC tissues, scale bar = 100 μm. b Volcano plot of DEGs in GSE101584.


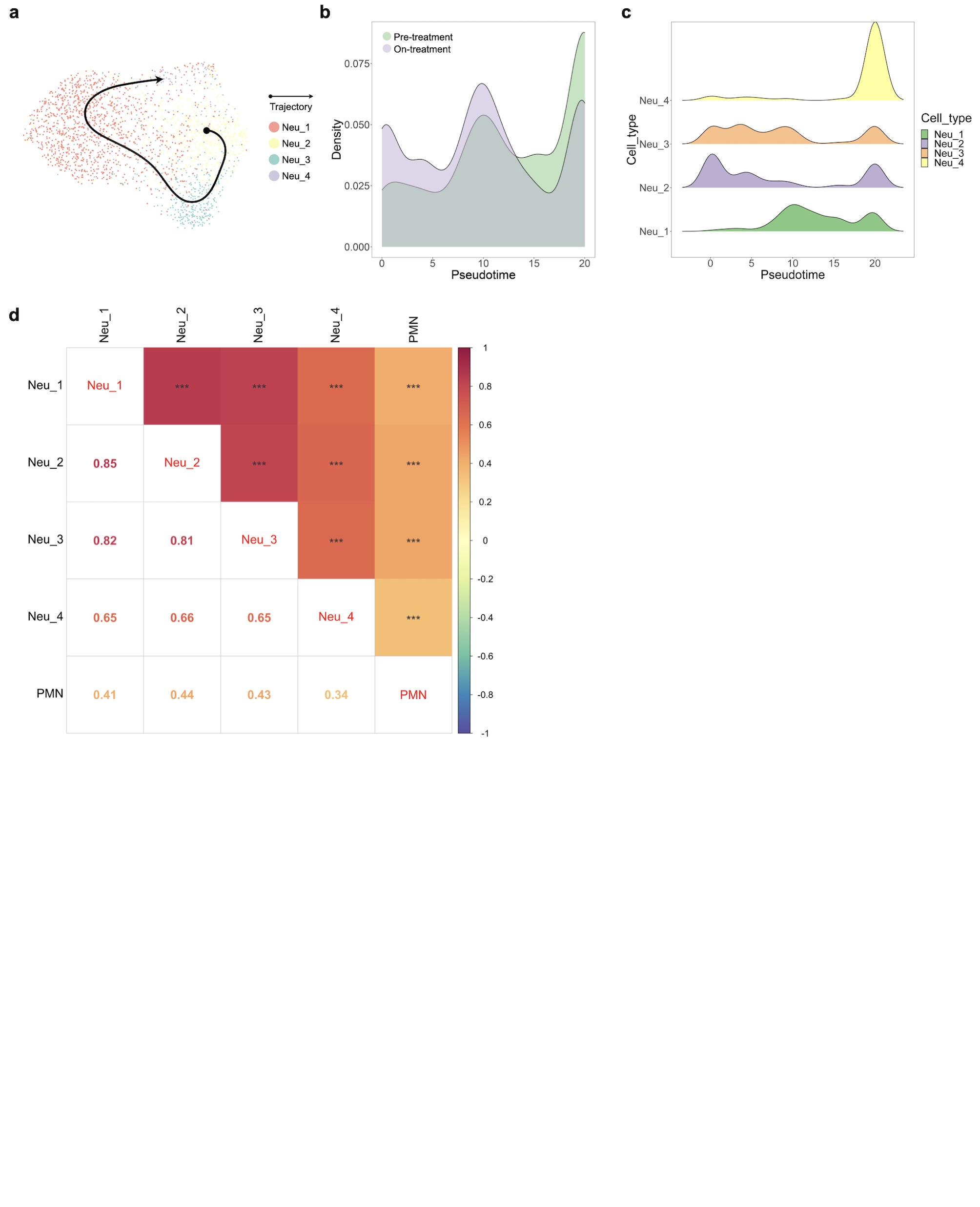


**Fig. S15** Neutrophil characterization, related to Fig. 4. **a** UMAP showing pseudotime inference of neutrophils. **b** The probability density plot of pre- and on-treatment neutrophils along the pseudotime. **c** The distribution of neutrophil subtypes along the pseudotime. **d** Heatmap showing the correlation between PMN gene expression and Neu subpopulation gene expression.


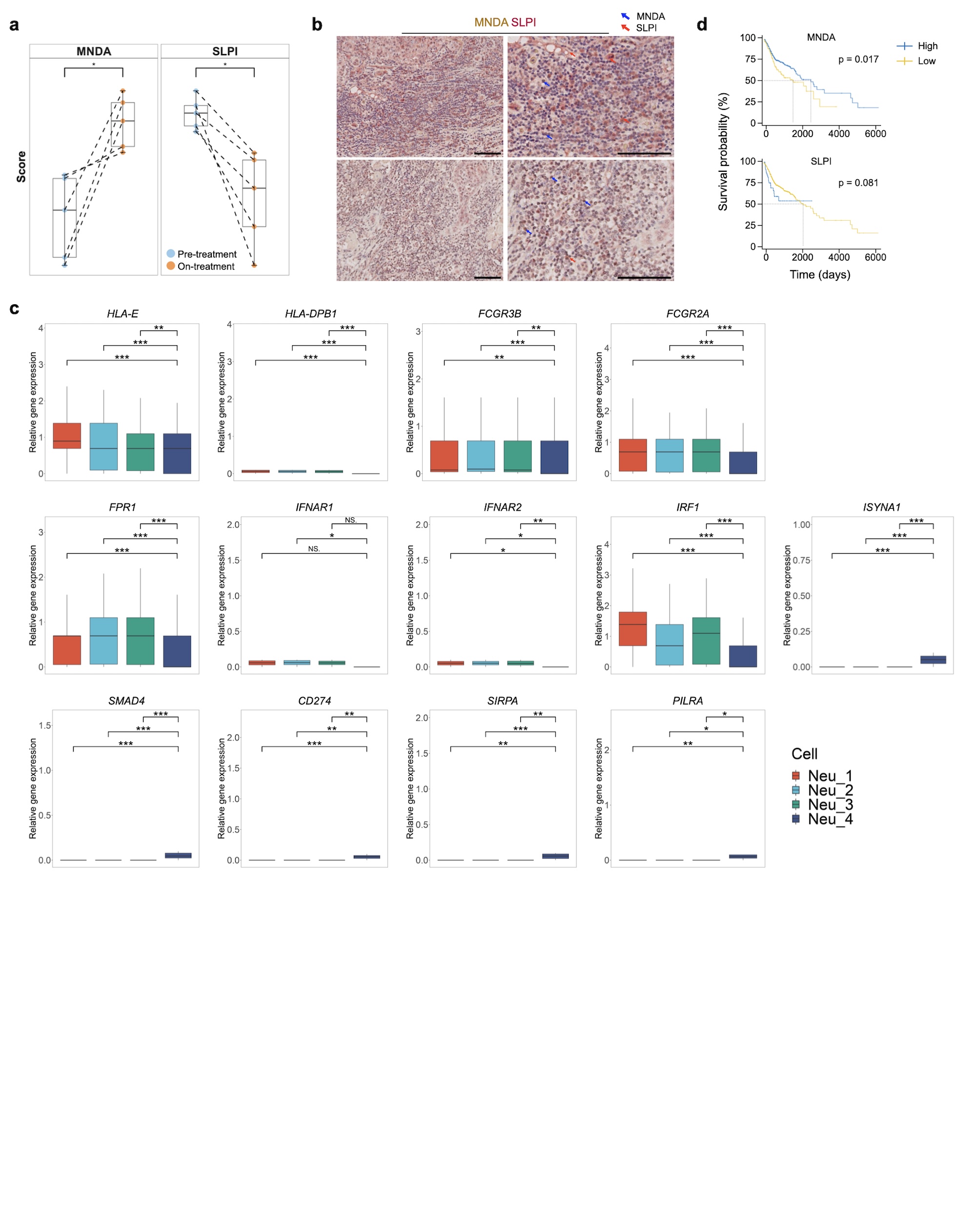


**Fig. S16** Neutrophil characterization, related to Fig. 4. **a** Box plot for quantitative analysis of IHC staining intensity of MNDA and SLPI. **b** Double IHC staining of MNDA and SLPI in LSCC tissues, scale bar = 100 μm. **c** Box plot for the expression of N1- and N2-TANs related genes in Fig. 4I in Neu_1 to Neu_4. A Kaplan-Meier curve was constructed to illustrate overall survival (OS). The mean values across groups were compared using a 2-tailed Student’s t-test. **d** Overall survival of *MNDA*^+^ TANs and *SLPI*^+^ TANs infiltration in TCGA HNSC cohort.


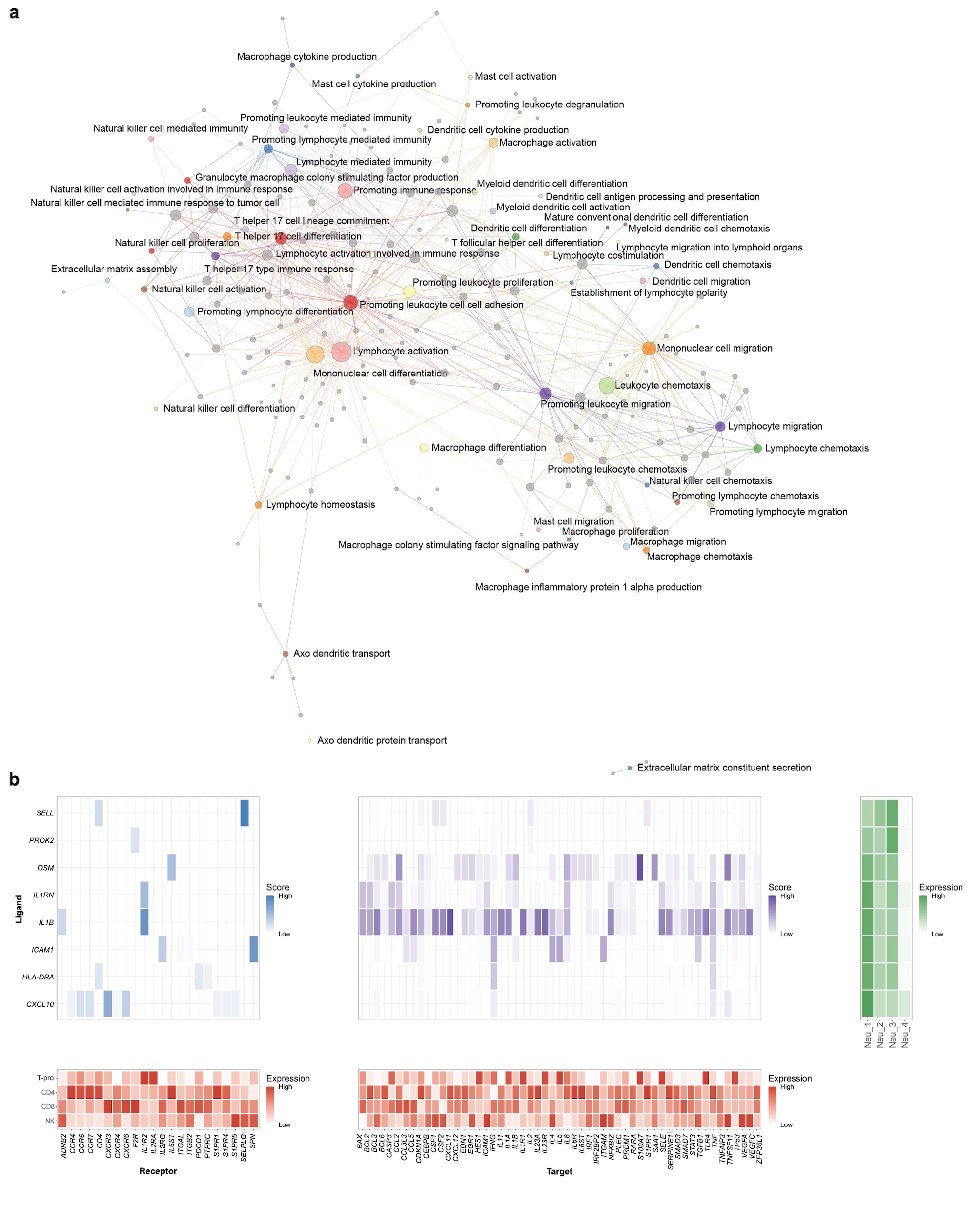


**Fig. S17** Interaction between neutrophil and T&NK cells, related to Fig. 5. **a** Network plot showing the functional enrichment of T&NK subsets genes regulated by TAN-N1 subsets. **b** Top, heatmap of scRNA-seq average interaction strength across neutrophil and T&NK subpopulations of top predicted ligands expressed by neutrophils that modulate T&NK cell types. Bottom, heatmap of scRNA-seq average expression of ligand-matched receptors and target genes expressed by T&NK cell types. Right, heatmap of scRNA-seq average expression of ligands expressed by Neutrophil cell types.


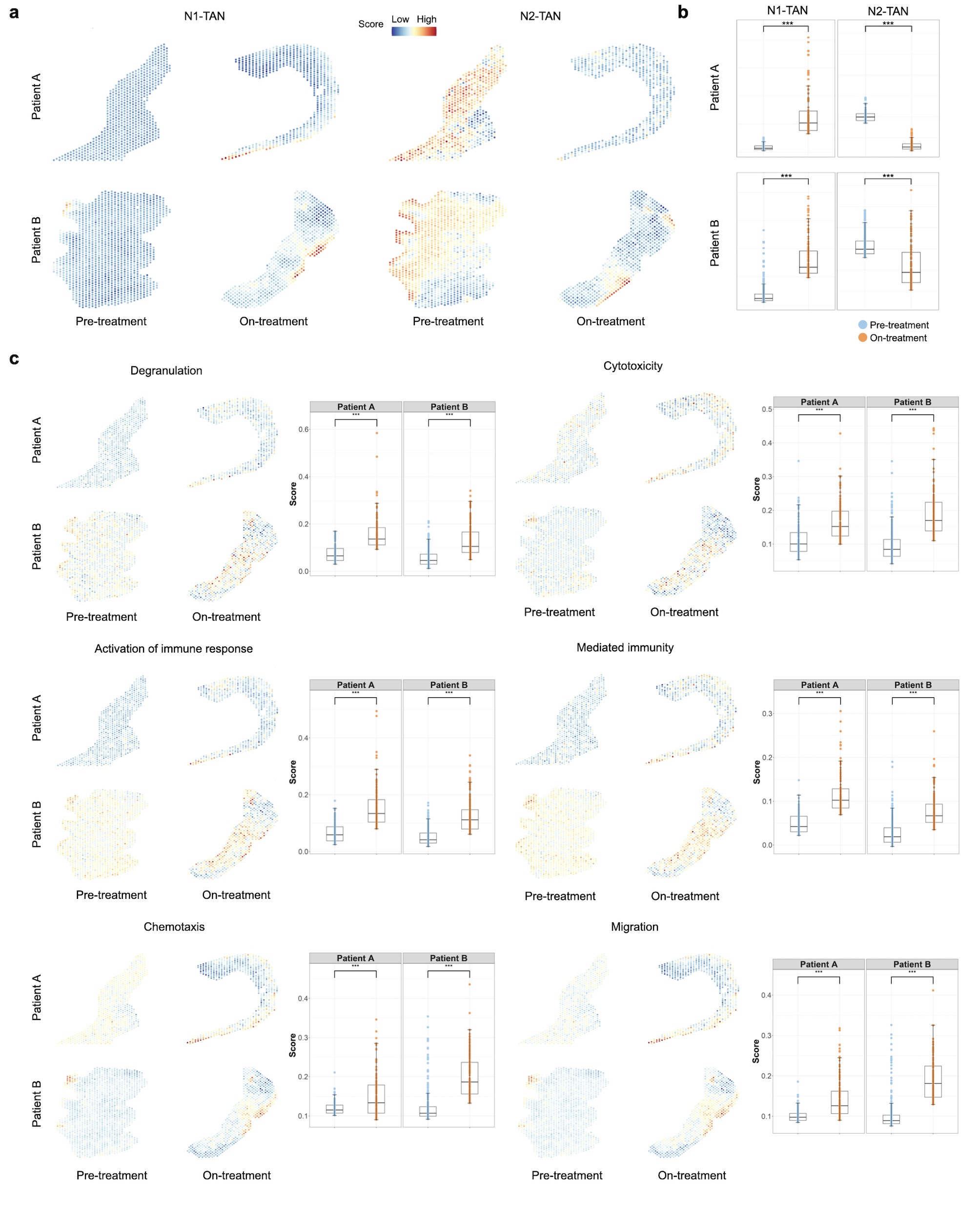


**Fig. S18** TAN characterization in TF, related to Fig. 5. **a** and **b** Spatial distribution of N1-TAN and N2-TAN in tumor front and box plots for quantitative analysis. **c** Activation score of TAN functions in tumor front and box plots for quantitative analysis. The mean values across groups were compared using a 2-tailed Student’s t-test.


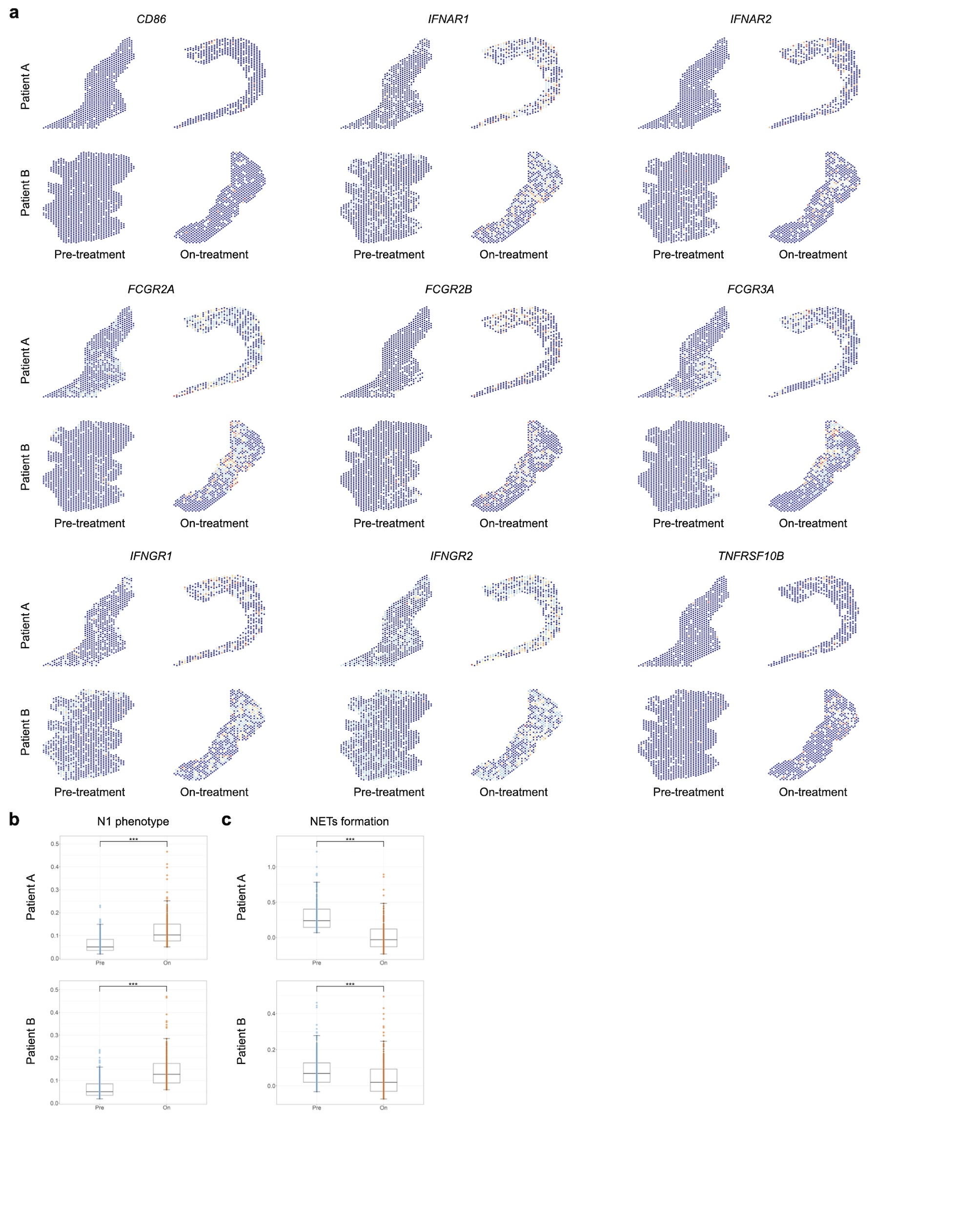


**Fig. S19** TAN characterization in TF, related to Fig. 5. **a** Expression intensity of N1-TAN functions-related genes in tumor front and box plots for quantitative analysis. **b** and **c** Box plots for quantitative analysis of expression intensity of N1 phenotype and NETs formation-related genes in TF. The mean values across groups were compared using a 2-tailed Student’s t-test


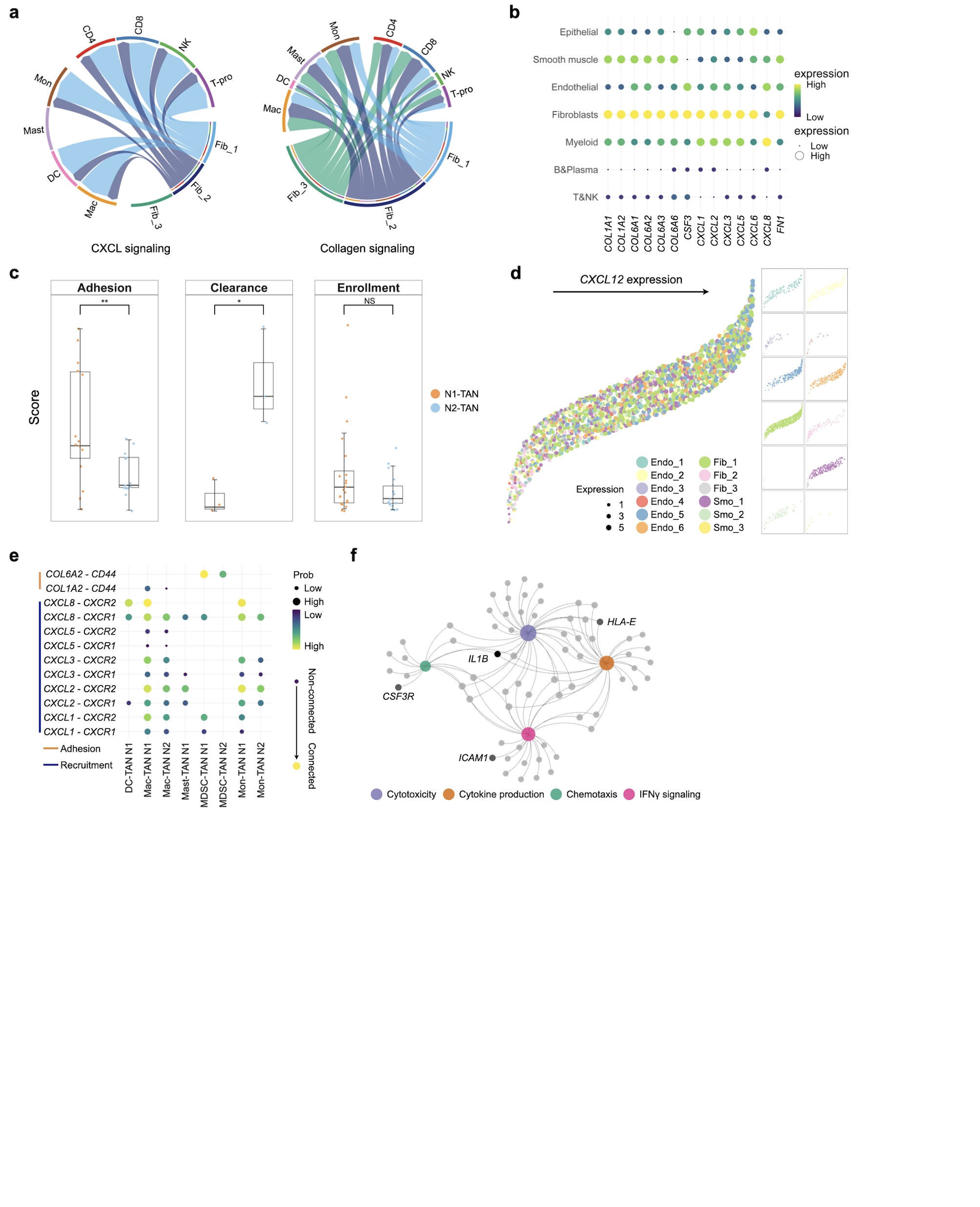


**Fig. S20** Interaction between neutrophil and other cells, related to Fig. 6. **a** chord diagrams showing fibroblasts recruit T&NK cells, macrophages, DC cells, and monocytes mainly through CXCL signaling pathway and promote their adhesion through collagen signaling pathway. **b** Dot plots of cell adhesion molecule and chemokine expression levels in seven major cell types. **c** Scores for fibroblast recruitment, adhesion and clearance of N1-TAN. **d** Dot plot showing the expression level of *CXCL12* in fibroblasts, endothelial cells, and smooth muscle cells. **e** Dot plot of the interaction of myeloid cells (DC, Mac, Mast, MDSC, and Mon) and TANs subclusters. **f** Network plot showing the functional enrichment of neutrophils subsets genes regulated by T&NK subsets. Each gray dot represents a gene and larger colored dots represent the enriched signaling pathways.


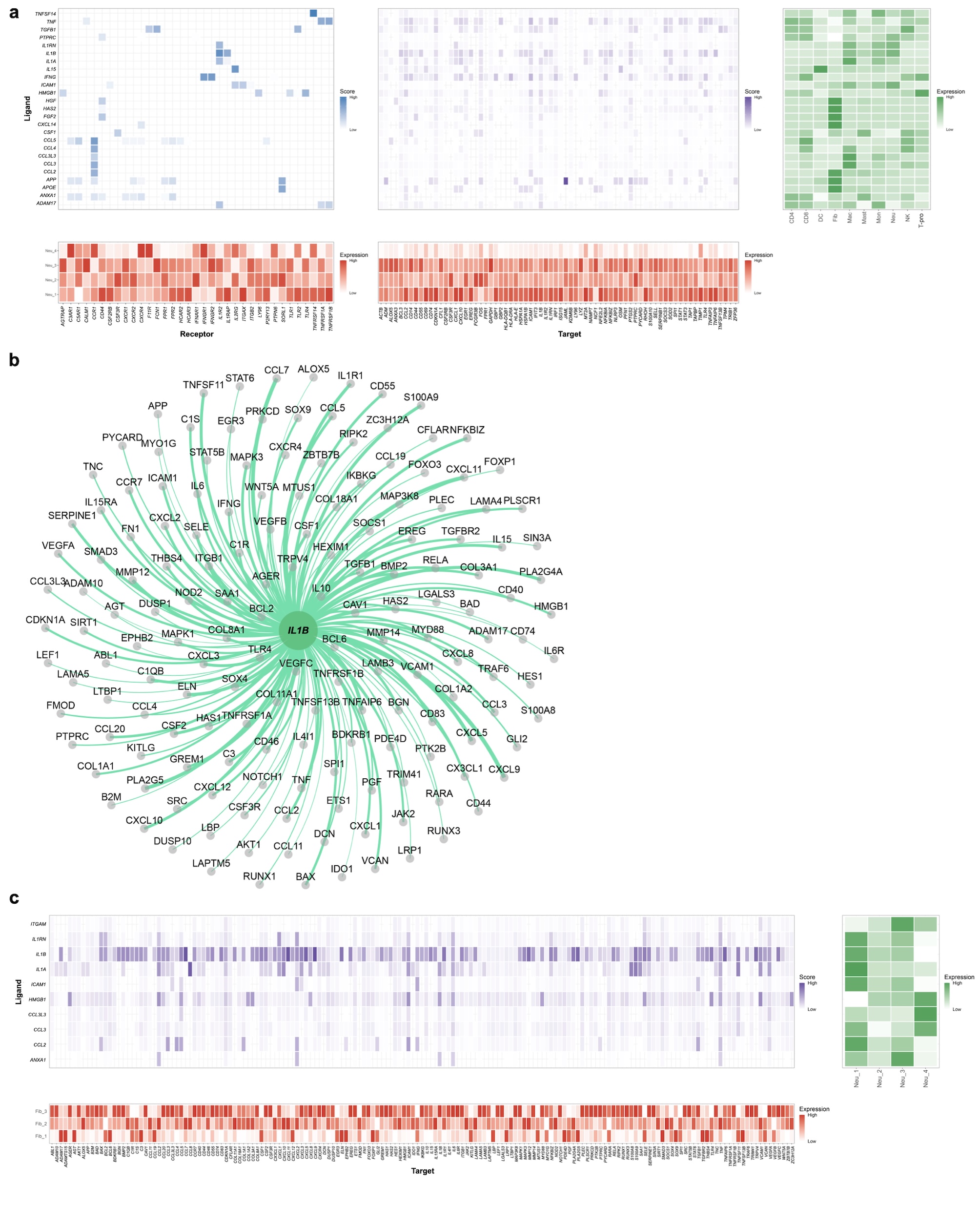


**Fig. S21** Interaction between neutrophil and other cells, related to Fig. 6. **a** Top, heatmap of scRNA-seq average interaction strength across immune cell subpopulations and neutrophils of top predicted ligands expressed by immune cells that modulate neutrophil. Bottom, heatmap of scRNA-seq average expression of ligand-matched receptors and target genes expressed by neutrophils. Right, heatmap of scRNA-seq average expression of ligands expressed by immune cells. **b** Regulatory network plot of *IL1B* expressed by neutrophil subclusters mediating target gene expression in fibroblasts subclusters. Each gray dot represents a target gene. **c** Top, heatmap of scRNA-seq average interaction strength across neutrophils and fibroblasts of top predicted ligands expressed by neutrophils that modulate fibroblasts. Bottom, heatmap of scRNA-seq average expression of ligand-matched target genes expressed by fibroblasts. Right, heatmap of scRNA-seq average expression of ligands expressed by neutrophils.


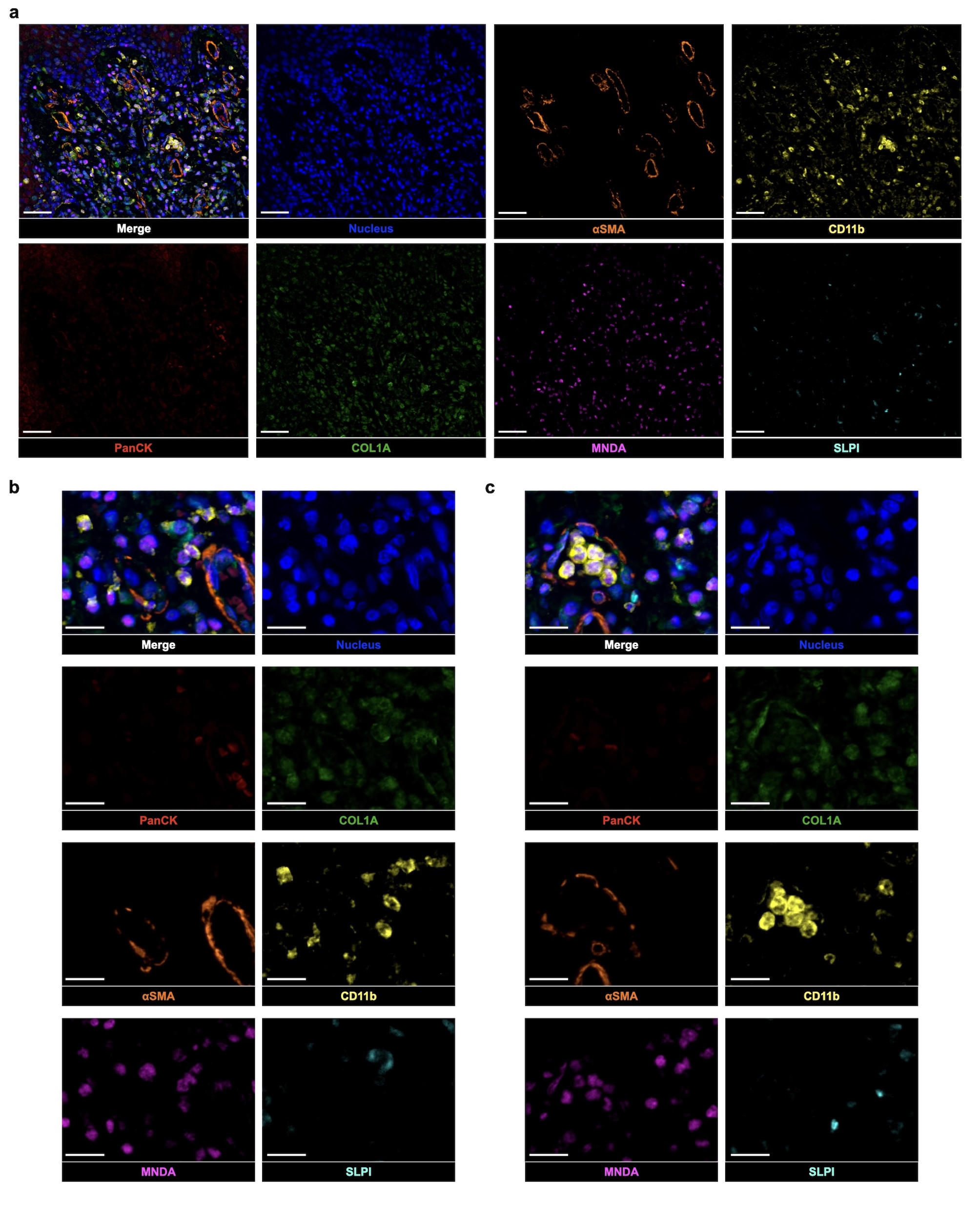


**Fig. S22** Interaction between neutrophil and other cells, related to Fig. 6. **a** mIHC staining of nucleus, PanCK, COL1A, αSMA, CD11b, MNDA, and SLPI in on-treatment tissues, scale bar = 50 μm. **b** and **c** mIHC staining of nucleus, PanCK, COL1A, αSMA, CD11b, MNDA, and SLPI in on-treatment tissues, scale bar = 20 μm.

**
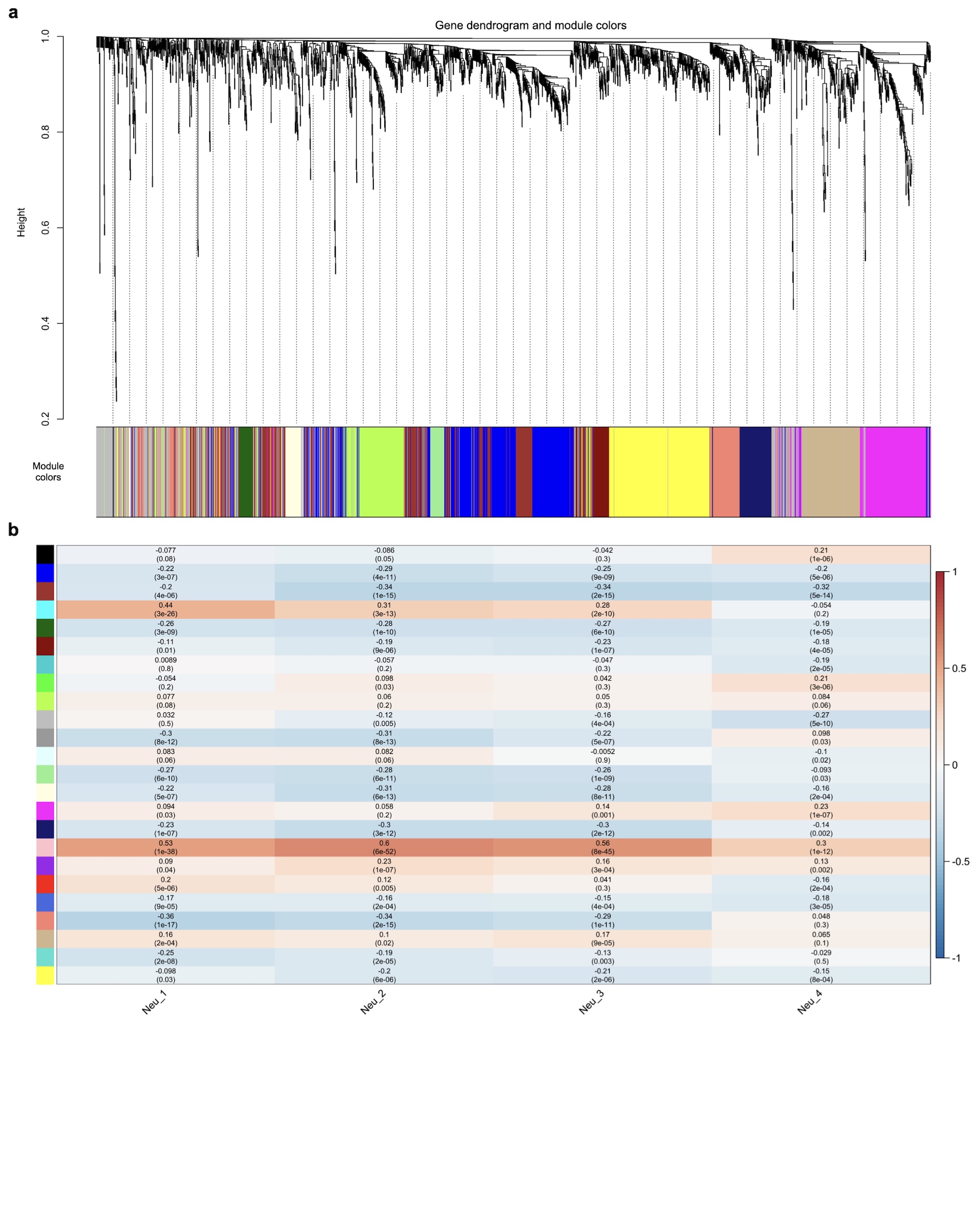
**

**Fig. S23** WGCNA analysis, related to Fig. 7. **a** Dendrogram of the WGCNA modules. **b** The correlation between eigengenes of each module and neutrophil infiltration.


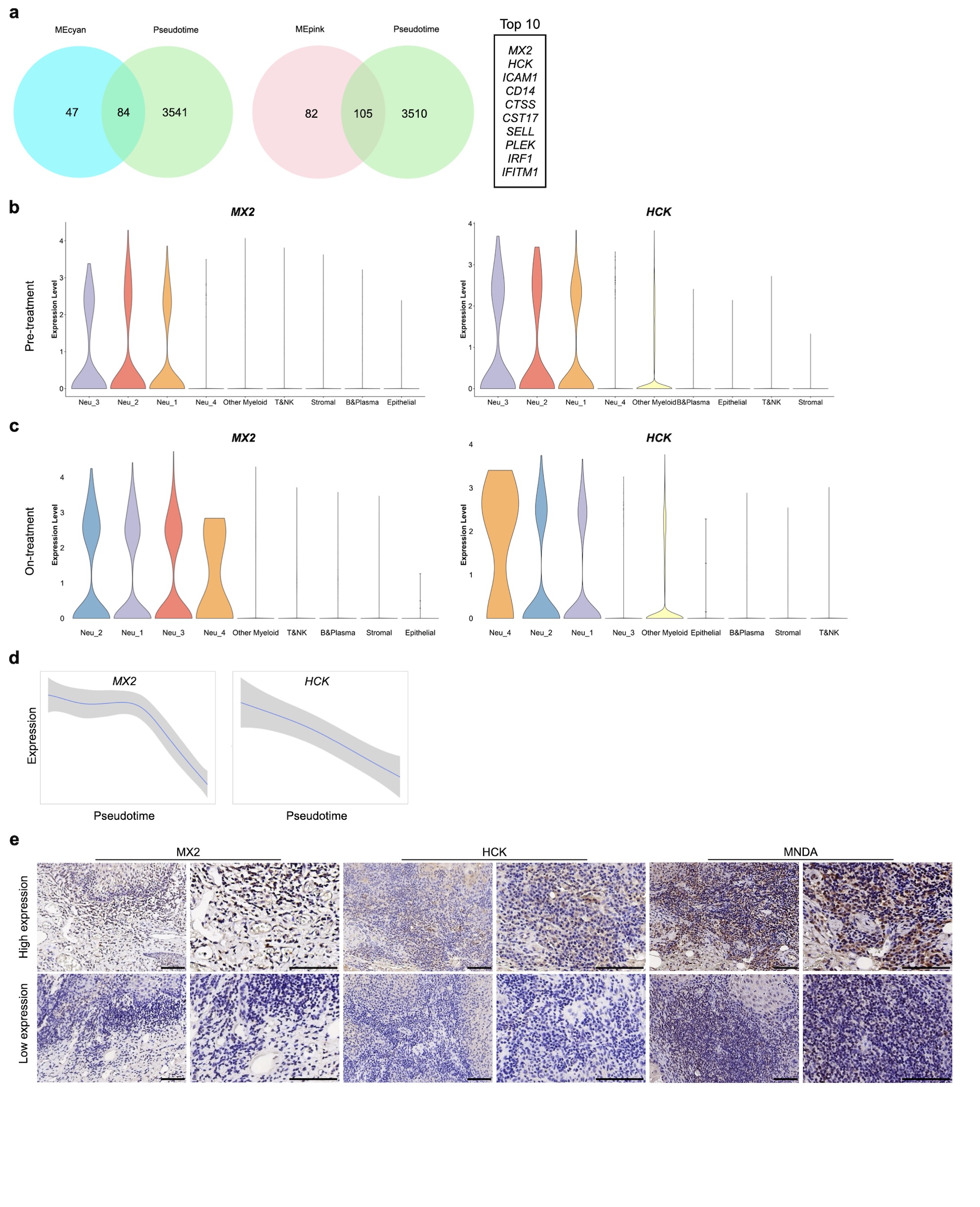


**Fig. S24** WGCNA analysis, related to Fig. 7. **a** Venn diagram of WGCNA modules and pseudotime trajectory-related genes. **b** and **c** Violin plots of expression level of MX2 and HCK of different cell types in pre-treatment and on-treatment samples, respectively. **d** Smooth curves showing the expression of MX2 and HCK along pseudotime. **e** IHC staining of MX2, HCK, and MNDA in pre-treatment LSCC tissues, scale bar = 100 μm.

Table S1. Patient scRNA-seq Data

| **Patient scRNA-seq Data** | | | | | |
| --- | --- | --- | --- | --- | --- |
| Patients | Samples | Estimated Number of Cells | Mean Reads/Cell | Median Genes/Cell | Median UMI/Cell |
| A | Pre-treatment | 4,665 | 111,562 | 1,179 | 2,964 |
| A | On-treatment | 7,988 | 58,542 | 1,492 | 3,771 |
| B | Pre-treatment | 4,408 | 22,360 | 615 | 1,241 |
| B | On-treatment | 6,305 | 17,287 | 655 | 1,234 |

**Table S2. Patient ST Data**

| **Patient ST Data** | | | | |
| --- | --- | --- | --- | --- |
| Patients | Samples | Spots | Median Genes/Spot | Median UMI/Spot |
| A | Pre-treatment | 4,590 | 1,730 | 3,518 |
| A | On-treatment | 1,937 | 2,644 | 10,655 |
| B | Pre-treatment | 2,600 | 1,814 | 5,417 |
| B | On-treatment | 1,640 | 2,154 | 6,767 |

**Table S3. Results of GO analysis on endothelial and smooth muscle cells upregulated genes linked to enhanced circulation and vascular permeability (on-treatment *vs* pre-treatment)**

| Description | Gene | Count | Cell |
| --- | --- | --- | --- |
| Blood vessel maturation | *S1PR1/CDH5/ACVRL1/ANKRD17* | 4 | Endothelial |
| Blood vessel endothelial cell migration | *THBS1/ID1/MMRN2/PIK3R3/FGF2/NRP1/ROBO1/NOS3/CDH5/FBXW7/MYH9/ATP2B4/PIK3C2A/HIF1A/ACVRL1/NUS1/ADAM17/FOXC2/ETS1/KDR/MIA3/NF1/STAT5A/MEOX2* | 24 | Endothelial |
| Vascular process in circulatory system | *BMPR2/ACTA2/LEPR/ECE1/DOCK4/TEK/INSR/TJP2/ADRA2A/SLC6A6/NOS3/CDH5/ROCK2/EDN1/ATP2B4/PIK3C2A/PLEC/ATP8A1/EDNRA/KCNJ8/GCLC/OCLN/ATP1B2/HRH2/FOXC2/SLC44A1/ITGA1/PLOD3/SLCO1C1/SLC7A2/ATP1A2/GRIP2/ABCC4/AVPR1A/SLC16A7/CPS1/KCNA5* | 37 | Endothelial |
| Regulation of blood circulation | *BMPR2/MDM2/EPAS1/ECE1/DOCK4/JAK2/ADRA2A/NOS3/CAMK2D/ZMPSTE24/EDN1/ATP2B4/PDE5A/FLNA/EDNRA/ATP2A2/HEY2/DSP/KCNE4/SLC8A3/AKAP9/EHD3/CELF2/ATP1B2/HRH2/PLN/TBX2/GJC1/DLG1/SMAD7/CACNA2D1/SCN4B/ATP1A2/DMD/TGFB2/RNF207/SHOX2/TMEM161B/AVPR1A/KCNA5/CACNA1H* | 41 | Endothelial |
| Positive regulation of vascular permeability | *PTP4A3/ANGPT1/APOE* | 3 | Smooth muscle |
| Regulation of blood circulation | PLN/ADRA2A/CASQ2/PDE5A/KCNA5/EPAS1/DES/FXYD1/TBX18/ATP2B4/CTNNA3 | 11 | Smooth muscle |
| Vascular process in circulatory system | ADRA2A/ROCK1/KCNA5/PTP4A3/AKAP12/ANGPT1/KCNMB1/APOE/DRD1/ATP2B4/PIK3C2A/SLC7A2/SLC2A4 | 13 | Smooth muscle |

**Table S4. Genes used to score each individual cell**

| Co-inhibitory | *TIGIT, PDCD1, CD96, CTLA4, LAG3, HAVCR2, BTLA, KLRC1, ENTPD1, LAYN, ITGAE* |
| --- | --- |
| Co-stimulatory | *CD2, CD28, TNFRSF4, TNFRSF9, TNFRSF18, ICOS, CD27, CD40LG* |
| Effector/cytotoxic | *CCL5, IL32, IFNG, PRF1, GZMA, GZMB, GZMH, GZMK, GNLY, NKG7, CXCR4, CXCR6* |
| N1 phenotype | *TNFSF4, CD86, CD80, TNFRSF9, FCGR3A, FCGR2A, FCGR1A, FCGR2B, FCGR3B, TNFRSF10A, TNFRSF10B, IFNGR1, IFNGR2, IFNAR1, IFNAR2* |
| N1-TAN marker genes | *PROK2, IL1B, OSM, CXCR2, FCGR2A, FFAR2, IL1RN, FPR2, HCAR3, PLEK, CMTM2, FPR1, S100A12, BCL2A1, AQP9, MNDA, ACSL1, S100A9, TREM1, S100A8* |
| NET formation | *MPO, PADI4, ELANE, MMP9, HMGB1, H2AC20, H2AC18, H2AC21, H4C14, H4C6, H3C14* |

**Table S5. The clinicopathological characters of 17 recurrent LSCC patients**

| No. | Gender | Age  (year) | Local recurrence | Lymph node metastasis | Date of completion of MTC | The interval between MTC and relapse (month) |
| --- | --- | --- | --- | --- | --- | --- |
| 1 | Male | 42 | No | No | 2019/1/23 | - |
| 2 | Female | 40 | No | No | 2019/3/22 | - |
| 3 | Male | 54 | Yes | No | 2019/4/19 | 22 |
| 4 | Female | 54 | Yes | No | 2019/5/31 | 4 |
| 5 | Female | 60 | Yes | No | 2019/7/9 | 2 |
| 6 | Female | 53 | No | No | 2019/8/30 | - |
| 7 | Male | 75 | Yes | No | 2020/6/16 | 2 |
| 8 | Female | 65 | Yes | No | 2020/7/31 | 2 |
| 9 | Female | 65 | No | No | 2020/9/25 | - |
| 10 | Male | 65 | No | No | 2020/10/23 | - |
| 11 | Male | 56 | Yes | No | 2020/11/20 | 17 |
| 12 | Female | 67 | No | No | 2021/5/7 | - |
| 13 | Male | 73 | No | No | 2021/5/18 | - |
| 14 | Male | 56 | No | No | 2021/5/28 | - |
| 15 | Female | 54 | No | No | 2022/4/8 | - |
| 16 | Male | 32 | No | No | 2022/4/19 | - |
| 17 | Male | 81 | Yes | No | 2022/6/21 | 5 |

- means not applicable
